# Interkinetic nuclear migration and basal tethering facilitates post-mitotic daughter separation in intestinal organoids

**DOI:** 10.1242/jcs.211656

**Published:** 2017-11-15

**Authors:** Thomas D. Carroll, Alistair J. Langlands, James M. Osborne, Ian P. Newton, Paul L. Appleton, Inke Näthke

**Affiliations:** 1 Cell & Developmental Biology, University of Dundee, Dundee DD1 5EH, UK; 2 School of Mathematics and Statistics, University of Melbourne, Melbourne 3010, Australia; 3 Dundee Imaging Facility, University of Dundee, Dundee DD1 5EH, UK

**Keywords:** Adenomatous polyposis coli, Intestinal epithelium, Interkinetic nuclear migration, Mitosis

## Abstract

Homeostasis of renewing tissues requires balanced proliferation, differentiation and movement. This is particularly important in the intestinal epithelium where lineage tracing suggests that stochastic differentiation choices are intricately coupled to the position of a cell relative to a niche. To determine how position is achieved, we followed proliferating cells in intestinal organoids and discovered that the behaviour of mitotic sisters predicted long-term positioning. We found that, normally, 70% of sisters remain neighbours, while 30% lose contact and separate after cytokinesis. These post-mitotic placements predict longer term differences in positions assumed by sisters: adjacent sisters reach similar positions over time; in a pair of separating sisters, one remains close to its birthplace while the other is displaced upward. Computationally modelling crypt dynamics confirmed that post-mitotic separation leads to sisters reaching different compartments. We show that interkinetic nuclear migration, cell size and asymmetric tethering by a process extending from the basal side of cells contribute to separations. These processes are altered in adenomatous polyposis coli (*Apc*) mutant epithelia where separation is lost. We conclude that post-mitotic placement contributes to stochastic niche exit and, when defective, supports the clonal expansion of *Apc* mutant cells.

## Abstract (precis)

**Highlighted Article:** The long-term position of mitotic daughters in the intestinal epithelium correlates with their placement immediately after mitosis. An initial separation at birth, predicts more divergent positions later.

## INTRODUCTION

Fate choices of proliferating cells are critical for intestinal homeostasis. Lgr5-positive stem cells (SCs) in the intestinal crypt base must be regulated carefully to balance their maintenance with the production of transit-amplifying (TA) progenitors that can specialise. Similarly, exit of TA progenitors from their proliferative niche has to be regulated to produce the appropriate number of post-mitotic differentiated cells. In the crypt, the position of cells relative to two niches, the stem cell and transit-amplifying compartments, reflects their fate (Ritsma et al., 2014). Accordingly, stem and transit-amplifying compartments differ in composition. The principal components of the intestinal SC niche are Paneth cells. Together with the surrounding mesenchyme, they provide Notch ligands, EGF and Wnts, which are critical for maintaining SCs, and this creates a local Wnt gradient along the intestinal crypt axis (Sato et al., 2011). Displacement of stem cells from Paneth cell contact causes serial dilution of membrane-bound Wnts, contributing to loss of stemness (Farin et al., 2016). Neutral competition for niche access by the 12–16 SCs in the crypt base governs net contraction and expansion of clones, leading to mono-clonal crypts over time (Lopez-Garcia et al., 2010; Snippert et al., 2010). Stem cells near the border of the stem cell niche are more likely to enter the transit-amplifying compartment and lose stemness. Stem cells residing at or near the crypt base are more likely to retain stemness (Ritsma et al., 2014). Traversing the transit-amplifying compartment is similarly accompanied by exposure to progressively less Wnt and other growth factors. Exit from this niche causes cell cycle exit. Such direct links between cell positioning and a graded niche signalling also operate in *Drosophila* (Reilein et al., 2017). These observations suggest that, in intestinal crypts, position, not the segregation of fate determinants, regulates cell fate.

Tissue homeostasis is perturbed in intestinal crypts mutant for key tumour suppressors such as adenomatous polyposis coli (*Apc*), KRAS, p53 (also known as TP53) and SMAD4. These mutations provide cells with a selective advantage and increase their ability to colonise proliferative niches. Measuring the competitive advantage of cells carrying these mutations using sophisticated lineage tracing experiments demonstrated a competitive advantage over wild-type cells that allowed their preferential retention in the proliferative niche (Vermeulen et al., 2013; Song et al., 2014). The expansion of such mutant clones is thought to underpin field cancerisation, the preconditioning of large tissue regions to neoplasia (Slaughter et al., 1953).

Our knowledge about cellular mechanisms that control cell positioning in the intestinal epithelium is limited, as is our understanding about how changes in such mechanisms can drive retention of mutant clones. Computational modelling suggests that the magnitude of the Wnt stimulus received at birth is a deciding factor for proliferative fate (Dunn et al., 2016). That suggests that decisions about cell position are set at birth. To test this hypothesis, we investigated daughter cell positioning along the crypt axis in 3D using intestinal organoids.

## RESULTS

**Fig. 1.**
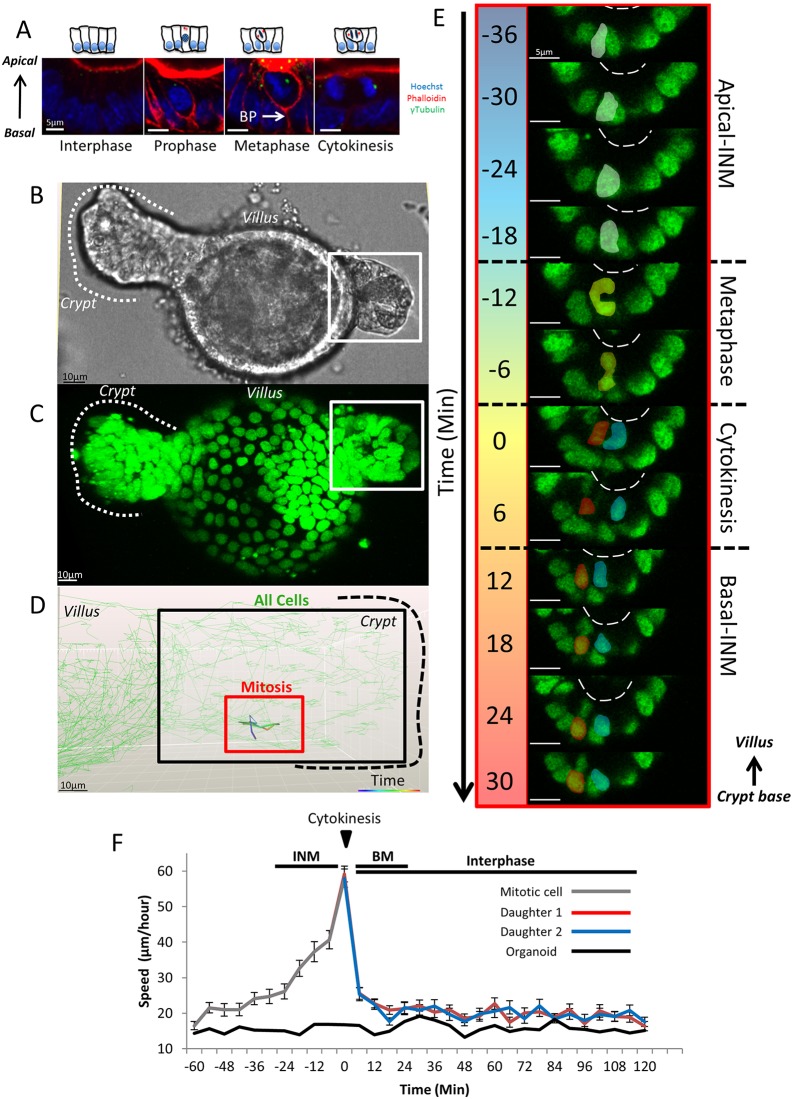
**Dynamics of mitosis in H2B–GFP-expressing** **intestinal organoids.** (A) Confocal sections of mitotic stages in intestinal organoids. Organoids were stained with Hoechst 33342, phalloidin and γ-tubulin (see also Fig. S1). The basal process (BP) is highlighted. Representative bright-field (B) and fluorescent (C) images of a wild-type H2B–GFP-expressing intestinal organoid after 24 h of doxycycline treatment. The positions of the crypt and villus domains of the organoid are indicated. (D) Manual tracking of a mitotic cell and its daughters. The track is colour-coded based on time (red box) and is overlaid onto the tracks of neighbouring cells (green), tracked automatically. In this example, tracks represent a time-lapse covering 66 min. The location of the crypt (outlined by the black dashed line) and villus domains are indicated. (E) Dynamics of mitosis in intestinal organoids. Sections (2D) of the mitotic cell highlighted in B. Prophase (white), metaphase (purple), cytokinesis (red) and daughter cell nuclei (blue and red) are shown. The estimated position of the apical surface is indicated by the dashed line. (F) Cell speed before, during and after mitosis, measured for mother (grey line) and daughters (red/blue lines). Movement of the entire organoid was measured for reference by tracking the centre of the organoid over time (black line). The average speed was calculated for 60 mitotic cells from three different organoids. Data is displayed as mean±s.e.m. Time-points encompassing INM, cytokinesis, basal cell movement (BM) and interphase are highlighted.

We measured cell positioning during and after mitosis in intestinal organoids, a widely accepted physiological model of the intestinal epithelium (Sato et al., 2009; Fatehullah et al., 2016b). They contain epithelial domains that correspond to crypt–villus architecture *in vivo*, and contain a comparable cellular composition. Cell division (Fig. 1A; Fig. S1) and polarity appear to be identical to that *in vivo* (Fatehullah et al., 2013), making organoids an ideal model system to understand the dynamic behaviour of the intestinal epithelium at temporal and spatial resolution impossible to achieve in tissue *in vivo*.

### Interkinetic nuclear migration operates in all intestinal epithelial cells and facilitates placement of mitotic sisters cells into different positions

Mitotic cells in the intestinal epithelium are easily distinguished (Fig. 1A; Fig. S1). During interphase, nuclei are positioned basally. Upon entering mitosis, interkinetic nuclear migration (INM) causes nuclei to migrate apically towards the centrosome, similar to what occurs in mitoses in the neuro-epithelium (Spear and Erickson, 2012). During this process, mitotic cells lose their columnar cell shape, become rounded and assume a position in the top half of the epithelial layer. Adjacent interphase cells expand into the basal space that is vacated by the migrating nuclei. Once INM is complete, spindles form and mitosis proceeds. After cytokinesis, newly formed cells move their nuclei basally and eventually assume a columnar shape. As mitotic cells round up, their apical surface remains aligned with that of the epithelial layer and they remain attached to the basement membrane by a process that extends from the basal side of cells, hereafter referred to as a ‘basal process’. Centrosomes are located apically in interphase cells and align laterally with condensed chromosomes during metaphase. These mitotic stages are indistinguishable between tissue and organoids (Fig. S1).

### Dynamics of INM during mitosis

The distinct movement of pre- and post-mitotic nuclei in intestinal epithelium is similar to INM in other tissues, where it has been implicated in cell fate decisions (Spear and Erickson, 2012). For instance, in the neuro-epithelium, INM facilitates differentiation by moving nuclei along apical-basal signalling gradients (Del Bene et al., 2008). In the developing fetal intestinal epithelium, INM has been implicated in the growth of epithelial girth (Grosse et al., 2011). The contribution of INM to intestinal homeostasis has never been examined. We examined how INM affects placement of mitotic sisters by tracking individual cells and their progeny during mitosis.

We directly monitored the position of mother and daughters during mitosis and after cytokinesis by performing live imaging of intestinal organoids expressing Histone2B–GFP (H2B–GFP). All nuclei in organoids derived from *H2B–GFP* mice robustly express GFP at 24 h after exposure to doxycycline allowing nuclear position to be used as a surrogate measure for cell position (Fig. 1B,C; Movie 1; Foudi et al., 2009). Measuring cell position in organoids required tracking cells in three-dimensional (3D) space. Techniques for accurately tracking cells in 3D are limited and we were unable to reliably track GFP-positive nuclei by using automated methods. Therefore, daughter cell behaviour was recorded manually by tracking cells using Imaris (Bitplane) (Fig. 1D).

Recordings revealed novel dynamic data about cell behaviour during mitosis. Mitosis lasted ∼60 min. Prophase was characterised by nuclear condensation and INM, followed by rapid formation of the metaphase plate. After spindle alignment and cytokinesis, both daughters slowly migrate basally until their nuclei align with adjacent interphase cells (Fig. 1E). During interphase, nuclei moved ∼25 μm/h in crypts, which increased to 60 μm/h during INM. Their speed during the basal cell movement was comparable to that in interphase, suggesting that INM is an active process and that the basal movement is passive (Fig. 1F).

### Daughter cells either remain adjacent or are separated from one another after mitosis

**Fig. 2.**
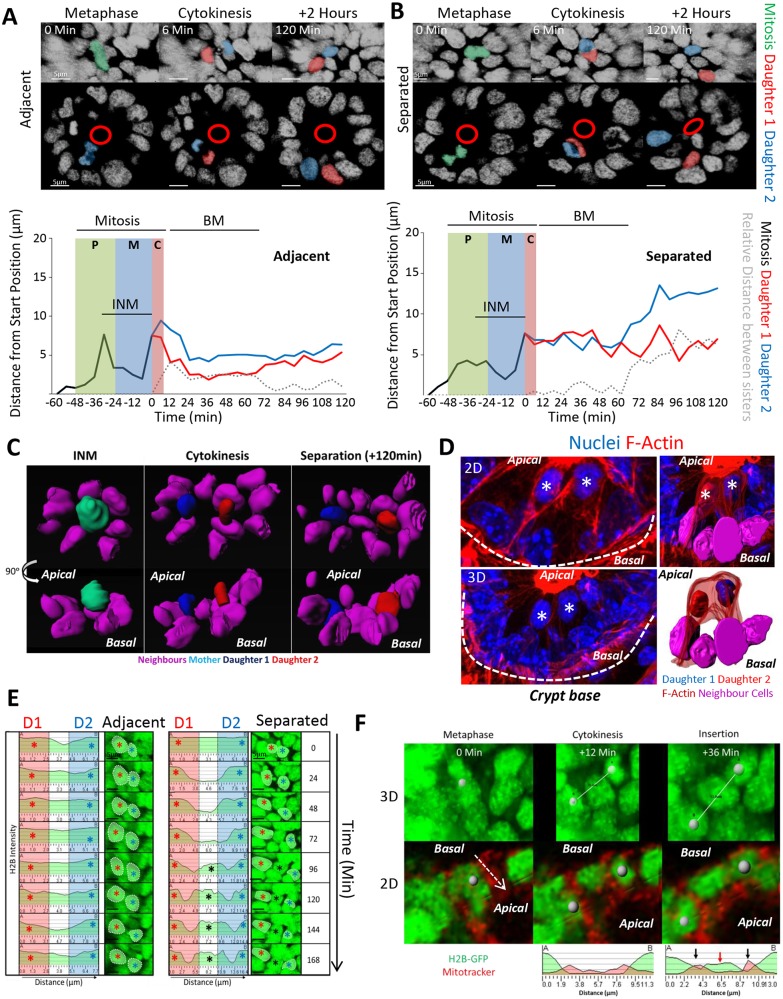
**Post-mitotic separation of daughter cells.** Mitotic cells were tracked manually for 60 min prior to cytokinesis and daughters for a further 120 min. Two types of mitotic types were revealed: (A) Daughter cells positioned adjacent or (B) that separated after mitosis. Displayed are 3D projections (top panels) and 2D sections through an organoid branch. Metaphase (green) and daughters (red/blue) are shown along with the approximate position of the apical surface (red circles). Representative tracks show the distance of the mitotic mother (black line) and daughters (red/blue lines) from the original starting position. Prophase (P), metaphase (M), cytokinesis (C), INM and basal cell movement (BM) are indicated. Distances between adjacently placed daughters (grey dashed line) are ≤1 nuclear width (6 μm) whereas distances between separating daughters are greater. (C) 3D rendering of neighbouring nuclei (purple), mother (cyan) and daughters (red/blue) for a post-mitotic separation event. Displayed are rotated views of cells and their direct neighbours at time-points encompassing INM, cytokinesis and after separation (120 min after cytokinesis). (D) Daughter separation occurs *in vivo*. Representative image of daughters at a crypt base. Samples were stained with Hoechst 33342 (blue) and phalloidin (red). Highlighted are two prospective daughters (white stars) displayed in 2D and 3D views (left panels). The dashed line represents the outline of the crypt base. Surface rendering (right panels) highlights cell–cell boundaries and neighbouring cell nuclei. The appearance of the chromatin and the proximity and position of the cells relative to each other are consistent with the highlighted cells representing mitosis in late telophase, producing two daughter cells. (E) H2B–GFP line-intensity profiles were created along a line connecting the centres of sister nuclei (red and blue asterisks) at indicated times after cytokinesis (=time 0). Reference images (3D projections) are displayed. Please note that the scaling for the *x*-axis, indicating distance, changes in the right-hand panel to accommodate the increased space between the separating daughters. (F) Individual frames of an H2B–GFP organoid stained with Mitotracker highlighting a mitotic cell whose daughters separate shortly after mitosis (grey spheres). Time-points reflecting metaphase, cytokinesis and the return of daughters to their interphase position (reinsertion) are shown. A line-intensity profile was generated between marked daughters (A and B) during cytokinesis and after ‘insertion’. After reinsertion, a discrete H2B–GFP peak was detected that corresponded to the neighbouring cell that displaces the two daughters (red arrow). The neighbouring cell has two distinct Mitotracker peaks on either side of its H2B–GFP signal (black arrows).

Tracking mitotic cells revealed two distinct outcomes for mitotic sisters. They either remain adjacent (6.0±1.2 μm apart; mean±s.e.m.) and become neighbours (Fig. 2A; Movie 2), or they separate (12.9±2.8 μm apart) and exchange neighbours (Fig. 2B; Movie 3). Rendering mitoses in 4D confirmed separation of the latter type of daughter cells by a neighbouring cell (Fig. 2C; Movie 4). Importantly, we observed similar mitoses *in vivo* with one sister positioned significantly displaced from the other by neighbouring cells (Fig. 2D). This data suggests that post-mitotic separation occurs in native tissue and in organoids.

To determine when mitotic sisters separate, we measured when neighbouring cells first appeared between them. Specifically, we measured the H2B intensity across the line connecting the centre of sister nuclei to visualise nuclear boundaries (Fig. 2E). For adjacent sisters, the line intensity profile was unchanged over time, indicating that the two nuclei remained in close proximity. For post-mitotic separations, an additional peak appeared between the peaks representing each sister, indicating the insertion of a neighbouring cell between them. Insertion of neighbours occurred 72–120 min after cytokinesis, indicating that displacement occurred during basal cell movement (Fig. 2E). Live-imaging of the mitochondrial network, as visualised with Mitotracker, clearly showed distinct cells between mitotic sisters, further confirming their physical separation (Fig. 2F; Movie 5).

We found other situations that also favoured separation. Separation could be facilitated by the movement of daughters of other mitoses in the immediate vicinity (Fig. S2; Movie 6). Furthermore, separation was favoured when mitoses occurred next to Paneth cells. Paneth cells are more adherent and stiffer than neighbouring epithelial cells (Langlands et al., 2016) and this could force one daughter cell out of the way (Fig. S2; Movie 7).

### *Apc* mutation alters placement of daughter cells

**Fig. 3.**
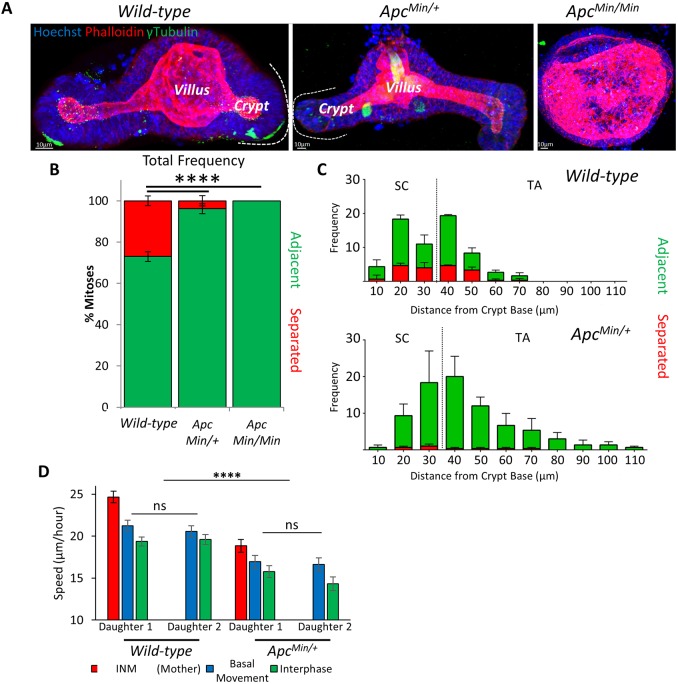
***Apc* mutant daughters separate less frequently.** (A) 3D projections of fixed organoids produced from small-intestinal crypts of wild-type and *Apc^Min/+^* mice stained with Hoechst 33342 (blue), phalloidin (red) and γ-tubulin (green). *Apc^Min/+^* organoids from cells that have undergone LOH form cysts (*Apc^Min/Min^*). The position of a crypt and vilus domain is marked for one branch (see also Fig. S3). (B) Types of mitotic daughter placement were scored in organoids (wild-type *n*=6, 491 mitoses; *Apc^Min/+^*, *n*=3, 227 mitoses; *Apc^Min/Min^*, *n*=7, 34 mitoses). Relative frequency of each type of mitosis was determined per organoid and averaged for replicate organoids. ****P<0.0001 for the number of adjacent and separating daughters between wild-type, *Apc^Min/+^* and *Apc^Min/Min^* organoids (*t*-test). (C) Mitotic cell position was determined relative to the crypt base for wild-type and *Apc^Min/+^* organoids. The frequency of each mitosis type along the crypt-villus axis was measured (*n*=3). The stem cell (SC) and transit-amplifying (TA) compartments are marked as defined by the average position of Lgr5–GFP-positive cells (see Fig. S5). Data is displayed as mean±s.e.m. (D) Nuclear speed was calculated in wild-type and *Apc^Min/+^* organoids (*n*=3 organoids, 20 cells). Data is displayed as the mean±s.e.m. speed for INM, basal cell movement and interphase. ****P<0.0001 for the speed of cells in wild-type and *Apc^Min/+^* organoids; ns, not significant (*t*-test).

APC is required for normal intestinal homeostasis, and mutations in *Apc* are common to most tumours in the colon (Fearnhead et al., 2001). The APC protein functions as a scaffold in Wnt signalling (McCartney and Näthke, 2008). It contributes to spindle orientation (Yamashita et al., 2003; Quyn et al., 2010) and cell migration along the crypt–villus axis (Nelson and Nathke, 2013). Lineage tracing and associated computational modelling has suggested that cells carrying *Apc* mutations are more likely to persist in intestinal crypts (Vermeulen et al., 2013; Song et al., 2014). To determine whether changes in the positioning of mitotic sisters could explain these observations, we isolated organoids derived from *Apc* heterozygous mice (*Apc^Min/+^*). These organoids are initially indistinguishable from wild-type organoids (Fatehullah et al., 2013) but transform into spherical, cyst-like structures (Fig. 3A) containing cells that have undergone loss of heterozygosity (LOH) (*Apc^Min/Min^*) (Germann et al., 2014). Mitoses appeared normal in *Apc^Min/+^* organoids; however, in *Apc^Min/Min^* organoids, abnormal mitoses with multipolar spindles and mitotic slippage were frequently observed (Fig. S3), similar to what is seen in cultured cells that lack APC (Dikovskaya et al., 2007). We compared the incidence of the two types of cell placements in wild-type *Apc^Min/+^* and *Apc^Min/Min^* organoids (Movie 1).

In wild-type epithelium, ∼30% of daughter cells separated whereas ∼70% remained adjacent (Fig. 3B). Separation was mainly associated with movement of neighbouring interphase cells during basal cell movement (72.8±17.7% of separations; mean±s.e.m.). Separation due to intercalation of other mitotic daughters in the immediate vicinity was less common (27.2±17.7% of separations). The frequency of the two mitotic types was equal in the stem and transit-amplifying compartments, suggesting that mitotic outcome is independent of cell position and type and can occur in any cycling cell that undergoes INM (Fig. 3C). To further confirm that mitotic separation is not specific to stem cells, we measured mitotic outcome in organoids treated with the GSK-3β inhibitor Chir99021 and the HDAC inhibitor valproic acid, which both increase the number of Lgr5-positive stem cells in the crypt (Yin et al., 2014). Treatment with Chir99021 and valproic acid did not significantly change post-mitotic separation of sisters (Fig. S4A,B), suggesting that the occurrence of post-mitotic separation is similar in all dividing cells along the crypt axis.

In *Apc^Min/+^* organoids there was a significant reduction in the frequency of post-mitotic separations. Sisters never separated in *Apc^Min/Min^* organoids (Fig. 3B). This suggests that *Apc* mutant sisters are more likely to remain adjacent to each other after division. There was also a significant overall reduction in cell movement between wild-type and *Apc^Min/+^* epithelial cells, including nuclear speed during INM (Fig. 3D), suggesting that cells remain adjacent because of reduced cell movement. We did not see any differences between compartments. Loss of post-mitotic separation was also induced by long-term treatment of organoids with high concentrations of Chir99021. This treatment caused organoids to grow as cysts, similar to *Apc^Min/Min^* organoids (Fig. S4). This suggests that hyperactive Wnt signalling induced either by *Apc* mutation or by GSK-3β inhibition can alter the frequency of post-mitotic separation, although it is possible that this is an indirect consequence of the changes in cells size and shape (see below).

### Post-mitotic separation of daughter cells directs niche exit

**Fig. 4.**
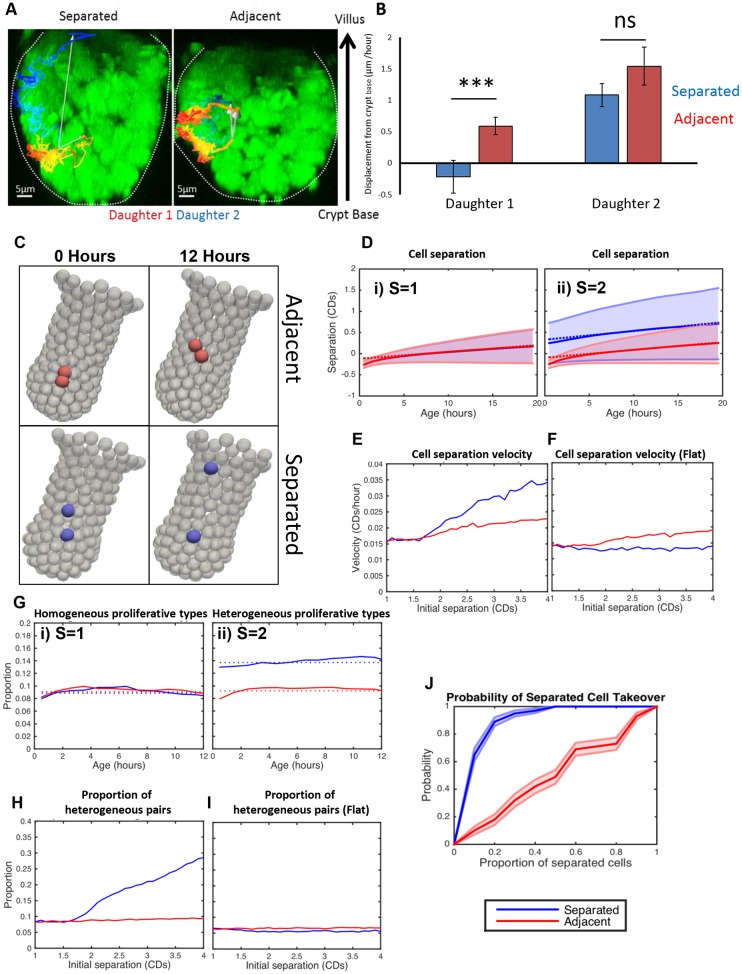
**Post-mitotic separation promotes both niche retention and exit.** (A) Representative images show examples of the long-term behaviour of daughter cells following separate or adjacent placement. An overlay of the track of daughters (daughter 1, red; daughter 2, blue) reveals the total displacement over the time course (white arrow). (B) Daughter cell position from the crypt base was measured after reinsertion into the epithelium (∼2 h) and at the final position able to be recorded (5–35 h). Displacement (movement of each daughter from the crypt base over time) was calculated for each daughter pair. Daughter 1 was defined as the daughter closest to the crypt base. Mean±s.e.m. values were calculated for separating (apart, *n*=28) or adjacent (together, n=84) sisters. ***P<0.001; ns, not significant (*t*-test). (C) Simulation results: representative images showing daughters initially placed adjacent to each other (red) and placed apart by one cell diameter (blue). Snapshots shown represent these situations immediately after mitosis and 12 h later (right hand panels). (D) Simulation results showing the distribution of cell separation as a function of time since birth. Results are shown for a homogenous population (i) of cell divisions where daughter cells are placed adjacent to each other (i.e. *S*=1 *CD* for all divisions), and for a heterogeneous population (ii) of cell divisions where *S*=1 *CD* for two thirds of divisions (red) and *S*=2 *CD*s for the remaining third (blue). The mean separation (solid line) and standard deviation (shaded region) is displayed. Linear fits to the distribution (from 5–15 h) are represented by dotted lines. CDs, cell diameters. (E) Simulation results for the effect of initial cell placement on separation velocity. For each separation, a heterogeneous population of divisions (two thirds with *S*=1 and a third with *S*≠1) is simulated and the corresponding separations (as shown in D) are calculated, and the distribution of values recorded. The separation velocity is calculated by taking the gradient of the linear fit to the mean of this distribution for both populations of divisions (adjacent in red and separated in blue). Cells initially placed further apart will separate more quickly than those placed together. (F) Same data as for E but with division restricted to the horizontal plane therefore removing the component of separation in the *z* direction. We see that when there is no *z* component to the division separation, the separation on division has no effect on the separation velocity. (G) Simulation results for the proportion of cell divisions that produce cells in different niches (i.e. one cell remains in the proliferative compartment while the other leaves) for simulations shown in D. Results are shown for a homogenous population (i) of cell divisions where all daughter cells are placed adjacent to each other (i.e. *S*=1 for all divisions) and for a heterogeneous population (ii) of cell divisions where *S*=1 for two thirds of divisions (red) and *S*=2 for the remaining third (blue). Constant fits to these distributions (using data for all ages) are denoted by dotted lines. (H) Simulation results showing how the average proportion of cell pairs with different positions (using the constant fit from F) depends on the initial separation. The same simulations were used as in E. Increasing separation leads to a larger proportion of cell pairs in different positions. (I) Same data as for H, but with division restricted to the horizontal plane therefore removing the component of separation in the *z* direction. We see that when there is no *z* component to the division separation, the separation on division has no effect on the proportion of cells in different niches. (J) Probability of separated cells taking over the crypt. Starting from the indicated proportion of separated cells, we run the simulation until all cells are ‘adjacent’ or ‘separated’; note that here separation on division is an inherited property (rather than being random as in all other simulations). The red line represents separated cells which are identical to adjacent cells, and the blue line shows results for when separated cells are four times more separated on division. This shows that separation is advantageous.

The two types of placements of mitotic sisters we discovered led to the hypothesis that post-mitotic separation allows differential exit of sisters from proliferative compartments. For instance, separation of stem cell daughters may increase the probability of one daughter to remaining in the stem cell niche compared to the other. Similarly, in the transit-amplifying compartment, post-mitotic separation could make it more likely for one daughter to remain in the proliferative compartment and the other to exit and terminally differentiate. To test this idea, we measured the distance of mitotic sisters from their starting positions and from each other after their birth and later. Shortly after cytokinesis, after both daughters had assumed their interphase position, regardless of mitosis type, one sister always remained near its starting position, whereas the other moved upward (Fig. 4A,B). At later times (up to 35 h after mitosis), differences between sisters were accentuated. If sisters had separated, one always remained close to its starting position while the other was displaced significantly upwards. In contrast, adjacently placed sisters were both displaced upwards (Fig. 4A,B). Thus, the initial difference in distance between sisters in the two types of mitoses was amplified over time, consistent with the idea that different placement of mitotic sisters can produce different outcomes for cell positioning.

To provide additional evidence for this idea, we used a previously established computational 3D model of intestinal crypts (Dunn et al., 2016) and asked whether post-mitotic separation could promote heterogeneous position/fate. To compare modelling results to our experimental data, simulations were performed with daughters placed adjacent to each other (as in previous computational models) or separated by a factor larger than a typical cell diameter. Simulations were performed using parameters derived from the primary data (see Materials and Methods). These simulations confirmed that post-mitotic separation often led to one daughter being retained close to its point of birth while the other was displaced upwards (Fig. 4C). There was a significant difference between the separation velocities between the two mitotic subtypes, indicating that daughters that initially separated moved apart much faster than those born adjacent to each other (Fig. 4D,E). In the model, the velocity increased only when daughters separated vertically (along the crypt axis) (Fig. 4E,F). Consistent with this data, greater retention tended to be observed when daughters separated vertically along the crypt axis (vertical separation ∼−0.77 μm/h; horizontal separation ∼−0.12 μm/h).

To test whether post-mitotic separation influences the number of heterogeneous cell pairs, we imposed a crypt-specific boundary separating a proliferative region from a non-proliferative region in the model. In this case, heterogeneous pairs are produced when one daughter is retained on the proliferative side of the boundary and the other exits. Consistent with our experimental results, simulations showed that separation led to more heterogeneous pairs than adjacent placements (Fig. 4G). The same results were produced for thresholds representing the TA–differentiated and SC–TA boundaries, which is similar to what has been reported in other studies (Vermeulen et al., 2013; Song et al., 2014). A greater separation distance at birth led to a higher number of heterogeneous pairs (Fig. 4H). Likewise, the increase in the number of heterogeneous pairs was dependent on vertical movement along the crypt axis (Fig. 4I). These data are consistent with our hypothesis that post-mitotic separation enhances divergent daughter fate by promoting the exit of one daughter from a niche while allowing the other to remain. Likewise, the model suggests that the clones retained through separation has a much higher probability of colonising the crypt than those remaining adjacent (Fig. 4J).

### Mechanisms for post-mitotic separation

Numerous mechanisms may be involved in post-mitotic separation. For instance, spindle orientation could direct placement of sisters, and we have indeed previously observed different types of spindle alignment in intestinal tissue (Quyn et al., 2010). To understand how mitotic placement is related to spindle orientation, we measured spindle alignment in organoids. Consistent with the previous data in whole tissue, we obtained some evidence for spindle orientation bias in the stem cell compartment of wild-type organoids. Although most spindles oriented parallel to the apical surface, as found in other reports (Bellis et al., 2012), we observed a small number of cells that oriented their spindles perpendicularly to the apical surface. There were no perpendicularly oriented spindles in the stem cell compartment in *Apc^Min/+^* organoids (Fig. S3). This is consistent with the idea that separating sisters can result from mitoses with perpendicularly aligned mitotic spindles. However, perpendicular spindle alignment was less frequent than the number of separating sisters, suggesting that additional processes are involved and that spindle orientation is not a reliable measure of post-mitotic separation.

### Basal tethering of daughter cells contributes to post-mitotic separation and is altered in *Apc* mutant organoids

**Fig. 5.**
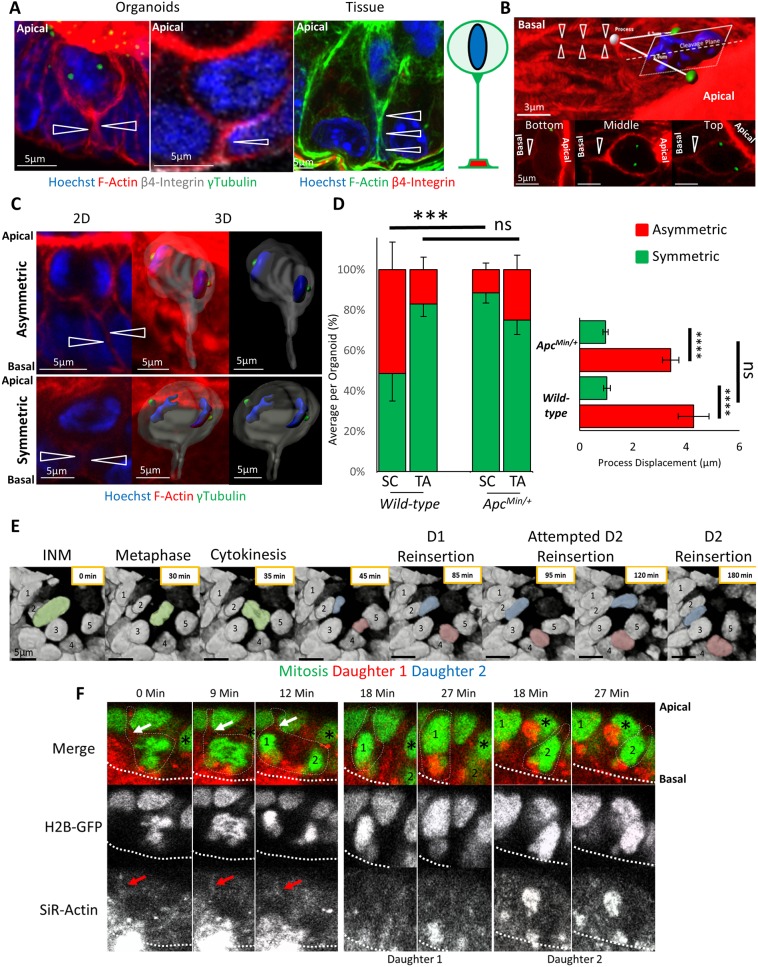
**Basal tethering of mitotic cells is altered in *Apc* mutant epithelia.** (A) 3D projections of mitotic cells in organoids and whole tissue reveal the basal processes (arrowheads). Samples were stained with Hoechst 33342 (blue), phalloidin (red), and for γ-tubulin (green) and β4-integrin (white). A schematic of the basal process is shown. (B) 3D projection of a mitotic cell aligned in metaphase showing the position of its basal process (arrowhead), centrosomes (green), nucleus (blue), and mitotic cleavage plane. Symmetric or asymmetric process inheritance was scored based on its placement relative to each centrosome. Accordingly, basal processes could be localised to the cell closer to the crypt base (‘bottom’), equidistant from each centrosome (‘middle’), or furthest from the crypt base (‘top’). In the displayed 2D sections, views were orientated with the crypt base towards the bottom in each image. (C) Process inheritance was scored by visual inspection of the position of basal processes relative to the mitotic cell in 3D. Two examples of mitotic cells are shown, one with asymmetric and one with symmetric process placement (see also Fig. S3). 3D surface rendering shows the position of the basal process. (D) Process segregation was scored by measuring the distance between the attachment point of the basal process and the centrosome of each prospective daughter in the stem cell (SC) and transit-amplifying (TA) compartments of wild-type (*n*=12 organoids, *n*=68 mitoses) and *Apc^Min/+^* (*n*=20 organoids, *n*=61 mitoses) organoids. Frequencies are displayed as the average percentage of each outcome per organoid and in each compartment. There was a significant reduction in the number of asymmetrically localised basal processes in the stem cell compartment in *Apc^Min/+^* organoids compared to in the stem cell compartment in wild-type organoids. Processes were scored manually and defined as asymmetric if significantly displaced from the cleavage furrow. To confirm manual scoring, process displacement was calculated for all scored asymmetric and symmetric processes. Displacement was defined as the difference between the distances from the process to each centrosome (right-hand panel). Data is displayed as mean±s.e.m. Process displacement in mitoses scored as asymmetric was significantly more common than in symmetric mitoses. ***P<0.001; ****P<0.0001; ns, not significant (*t*-test). (E) Individual frames of a time-lapse movie reveal the repeated attempt of one daughter (red) to assume the original position of the mother (green) while the other daughter (blue) moves on. (F) Individual frames of a time-lapse of H2B–GFP organoids stained with SiR-Actin showing a cell whose daughters undergo post-mitotic separation. Displayed are time-points encompassing metaphase (0 min), cytokinesis (9–12 min) and the two daughters during reinsertion (18-27 min), when they become separated by a neighbour (black asterisks). An asymmetric process (white/red arrows) is located closer to daughter 1 on one side of the putative cleavage furrow. The apical surface is denoted by the thick dashed line.

Another mechanism that may affect placement of daughters involves the basal process that tethers mitotic cells to the basement membrane (Fleming et al., 2007). This process is formed during INM and persists throughout metaphase. The basal process is rich in F-actin and is tethered at the basement membrane through β4-integrin (Fig. 5A)*.* Tethering of daughter cells after cytokinesis and during basal cell movement provides a direct means to guide daughters. Asymmetric tethering of mitotic cells has been proposed to facilitate anisotropic movement away from the stem cell compartment and can coincide with the segregation of planar cell polarity markers in the colonic epithelium (Bellis et al., 2012).

To determine whether asymmetric tethering of sisters operated in organoids and contributed to their placement, we measured the position of basal processes relative to prospective daughters. We distinguished whether the process was positioned symmetrically or asymmetrically. Processes attached close to the cleavage plane, equidistant to both centrosomes, were classified as symmetric. Those attached closer to one centrosome were classified as asymmetric. For asymmetrically placed processes, we also measured their position relative to the crypt base (i.e. whether the mitotic sister they were connected to was closer to the bottom or top of the crypt) (Fig. 5B,C). The basal process in all separating mitoses was significantly more displaced from the cleavage furrow than in adjacent mitoses (Fig. 5D). Accordingly, symmetrical processes that can be inherited by both daughters are predicted to facilitate adjacent cell placement (Fig. 5C, bottom panels), whereas asymmetric processes, which are inherited by only one daughter, predicts daughter separation (Fig. 5C, bottom panels).

The proportion of symmetrically and asymmetrically placed basal processes differed between the stem cell and transit-amplifying compartments. In the latter, the proportion of asymmetric basal processes was ∼20%, similar to the proportion of separating mitotic daughters. However, in the stem cell compartment, this number increased to 50% (Fig. 5D). This observation is consistent with previous results in colonic crypts (Bellis et al., 2012) and may be caused by differences in the architecture and resulting cell packing in the crypt base (Quyn et al., 2010). On the other hand, there was no significant difference in the relative number of post-mitotic separations between compartments (Fig. 3C). It is likely that asymmetric basal tethering is not sufficient for post-mitotic separation and that other mechanisms contribute.

In both regions, most asymmetrically positioned processes localised to the daughter cell closer to the crypt base, predicting that the untethered daughter was most likely to be displaced upwards. Asymmetrical basal process placement was a feature of mitotic cells with spindles that were perpendicularly aligned to the apical surface, suggesting that spindle orientation and basal process placement are linked (Fig. S3). Live imaging suggested that the basal process guides basal cell movement. The tethered daughter migrated basally to assume the interphase position of the mother, while the untethered daughter moved freely, allowing sister separation. This was particularly obvious when a daughter required multiple attempts to reintegrate into the epithelium (Fig. 5E; Movie 8). In *Apc^Min/+^* intestinal organoids fewer processes were placed asymmetrically, consistent with the significantly reduced frequency of separating sisters in *Apc^Min/+^* organoids (Fig. 5D). We propose that asymmetric processes facilitate the displacement of one daughter cell from the niche by allowing it to separate from its sister, rather than simply aiding in their retention (Bellis et al., 2012). To provide evidence for this hypothesis, we performed live imaging of H2B–GFP organoids treated with SiR-Actin (Lukinavicius et al., 2014). As expected, daughter separation correlated with asymmetric segregation of the basal process (Fig. 5F; Movie 9). We could not detect planar cell polarity markers or longitudinally oriented basal asymmetry described in isolated colonic crypts in intestinal organoids, (Bellis et al., 2012). It is likely that differences between colonic and intestinal epithelia and different sample preparation are responsible.

### *Apc* loss and hyperactive Wnt signalling restrict separation of sisters by inhibiting INM and changing cell size and morphology

**Fig. 6.**
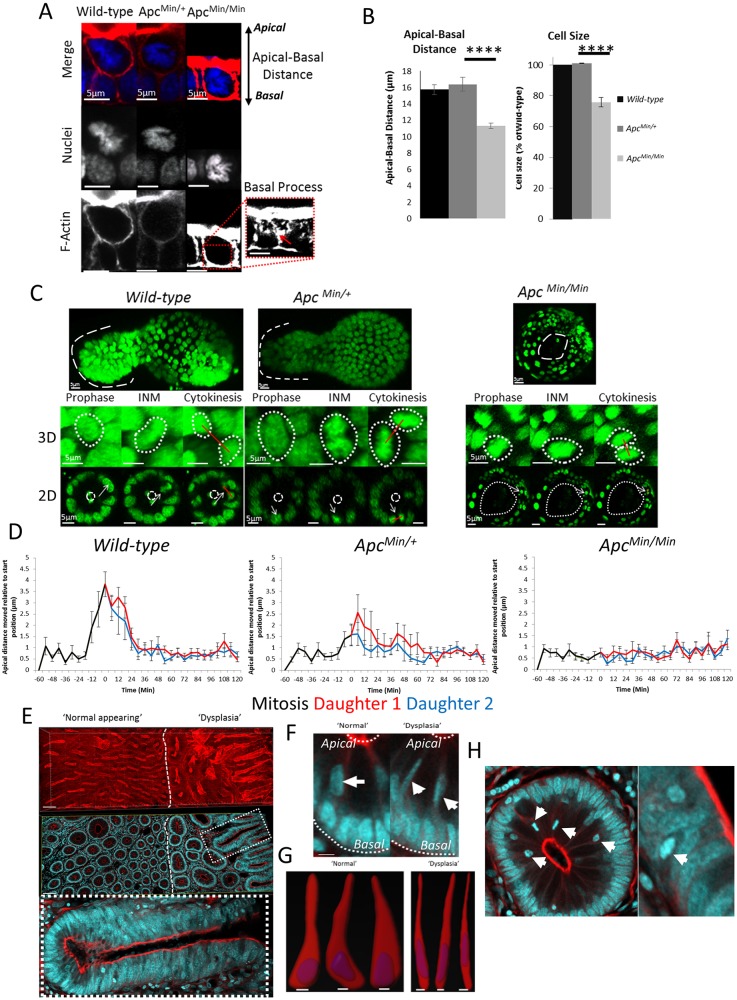
***Apc* mutation limits the ability of daughters to separate by preventing interkinetic nuclear migration and reducing cell size.** (A) Representative images of metaphases in wild-type, *Apc^Min/+^* and *Apc^Min/Min^* intestinal organoids stained with Hoechst 33342 (blue) and phalloidin (red). The red arrow points to the basal process. (B) The apical-basal distance of interphase cells was measured in wild-type, *Apc^Min/+^* and *Apc^Min/Min^* organoids in images (left panel). There was a significant difference in the apical-basal distance between wild-type and *Apc^Min/Min^* organoids. Cell size was measured in isolated wild-type, *Apc^Min/+^* and *Apc^Min/Min^* cells by using flow cytometry. The median forward scatter was determined for each genotype and averaged. Data are mean±s.e.m. (*n*=3) and are displayed relative to the size of wild-type cells. ****P<0.0001 for the difference between the relative cell size of wild-type and *Apc^Min/Min^* organoids (*t*-test). (C) Individual frames from an H2B–GFP organoid movies showing INM. For each genotype, a representative mitosis is shown at prophase, and during INM and cytokinesis. 3D (maximum intensity projections) and transverse (2D) views through an organoid branch or cyst are shown. Crypt domains are indicated in the upper panels by the dashed line. The estimated position of the apical surface is shown by the dashed circles in the lower panels. The white arrow in the middle and bottom panels points to an example of a mitotic cells that fails to undergo INM. (D) Dynamics of INM during mitosis in wild-type, *Apc^Min/+^* and *Apc^Min/Min^* was measured relative to the starting distance (*n*=10 cells per genotype). Data is displayed as mean±s.e.m. Measurements for mother (black line) and daughters (red and blue lines) are superimposed (see also Fig. S5). (E) A Vibratome section of human FAP colonic tissue stained with Hoechst 33342 (blue) and phalloidin (red). Displayed are 3D projections (top panel) and section views (bottom panel). Highlighted are regions of ‘normal-appearing’ and ‘dysplastic’ regions. The highlighted area (dashed line) shows a crypt with cell pile-ups/pseudo-stratification. (F) Magnified view of a crypt with ‘normal’ and ‘dysplastic’ regions of FAP colonic tissue from panel E. The basal and apical surfaces are highlighted by white dashed lines. Cells undergoing interkinetic nuclear migration are highlighted by white arrows. Scale bars: 10 μm. (G) Cell morphology of three cells from crypts in ‘normal’ and ‘dysplastic’ regions of FAP tissue. Displayed are surface renders of F-actin and nuclei for individual cells. Scale bars: 5 μm. (H) Examples of misplaced mitotic cells in FAP tissue. White arrows point to putative metaphase cells, which are abnormally localised a substantial distance away from the apical surface.

We did not detect sister cell separation in *Apc^Min/Min^* organoids. Instead, cells in metaphase usually lay in the plane of the epithelium in line with interphase nuclei and only had short compressed basal processes, which were difficult to visualise (Fig. 6A). In addition, cell morphology was altered and the distance between apical and basal surfaces was significantly reduced (by ∼25%) compared to the distance in wild-type or *Apc^Min/+^* cells (Fig. 6A). To determine whether this was due to changes in cell shape or overall cell size, we measured the volume of isolated single cells from wild-type, *Apc^Min/+^* and *Apc^Min/Min^* organoids using flow cytometry. There was no significant difference between wild-type and *Apc^Min/+^* cells, but cell size in *Apc^Min/Min^* organoids was reduced by 25%, indicating that a smaller cell volume was responsible for the reduced cell height (Fig. 6B). This suggests that, in cells lacking wild-type *Apc*, space restriction causes a reduction in apical-basal distance to prevent INM and restrict basal process formation, preventing post-mitotic separation.

To directly determine whether and how INM was altered in *Apc^Min/Min^* organoids, we first measured the distance of mitotic nuclei relative to the basal membrane of the epithelial layer in wild-type, *Apc^Min/+^* and *Apc^Min/Min^* organoids. The basal reference was established as the plane formed between the nuclear centres of neighbouring cells proximal to the mitotic/daughter cells (Fig. S5). In wild-type cells, the distance covered by INM was ∼4 μm (Fig. 6C,D). The distance covered during INM was not significantly shorter in *Apc^Min/+^* cells but the speed of nuclei during INM was significantly reduced (Fig. 6C,D). As expected, in *Apc^Min/Min^* organoids there was no apical displacement (i.e. no INM) and all daughters were placed adjacently. Similar results were achieved in organoids chronically treated with Chir99021 to hyperactivate Wnt signalling (Fig. S5). We also observed mitoses in *Apc^Min/+^* organoids that exhibited no INM. One possible explanation is that some cells in *Apc^Min/+^* organoids had already undergone LOH. The reduced nuclear movement during INM in *Apc^Min/+^* cells is likely to cause a decrease in the time mitotic cells spend near the apical surface. Taken together, these data suggest that INM is important for the ability of sisters to separate (Fig. 6C,D).

To corroborate these observations *in vivo*, we compared the morphology of interphase and mitotic cells in normal and transformed tissue isolated from a familial adenomatous polyposis (FAP) patient (Fig. 6E). There was a striking morphological change in cells from dysplastic regions. In contrast to what is seen in *Apc^Min/Min^* organoids, which displayed greatly reduced apical-basal distance, there was a significant lateral compression of cells in the human tissue samples (Fig. 6F,G) that correlated with the pseudo-stratification caused by ‘pile-ups’ of cells along the crypt–villus axis (Fig. 6E). This is consistent with a reduced cell size. However, in tissue, surrounding mesenchyme and/or adjacent crypts may cause lateral compression, whereas organoids are not mechanically restricted allowing cells to assume a more square shape. This lateral compression likely restricts the ability of nuclei to undergo INM and reach the apical surface and separate. Consistent with this idea, we found metaphase cells that were abnormally distant from the apical surface in these tissue areas (Fig. 6H). This is predicted to result in decreased post-mitotic separation and may contribute to the observed pseudo-stratification and cell overcrowding in crypts.

## DISCUSSION

**Fig. 7.**
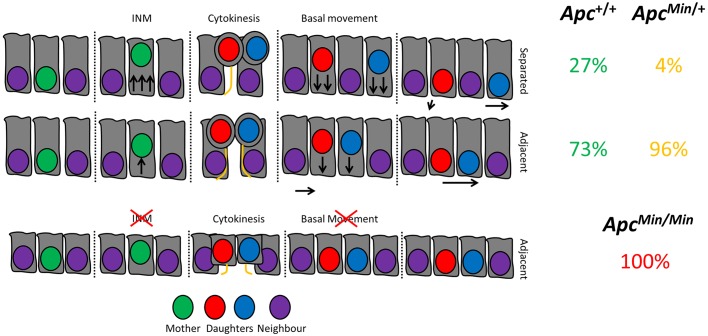
**Model for how prolonged niche retention in *Apc* mutant cells arises.** In untransformed intestinal epithelia, normal tissue architecture is maintained. During mitosis, cells undergo INM, characterised by the apical movement of nuclei as the cell rounds up. This permits daughter cells to remain proximal or to become displaced from one another by neighbouring cells. Displacement promotes retention of a cell at its birthplace and allows the other to exit the niche. This displacement is facilitated by INM and the asymmetric segregation of the basal process. After mutation of one *Apc* allele (*Apc^Min/+^*), mitoses become biased towards adjacent placement and migrate slower, facilitating niche retention. Upon loss of heterozygosity (*Apc^Min/Min^*), all cells lose the capacity for separation due to reduced cell size and inhibited INM. As a result, symmetrical cyst growth could be promoted, promoting altered tissue architecture.

Where a cell is born is linked to its identity. In this study, we show that daughter cells can separate immediately after cytokinesis and assume increasingly diverging positions over time (Fig. 7). This means that one sister is more likely to exit a compartment where it was born than the other. For stem cells, this means that one sister is more likely to differentiate into a progenitor. For transit-amplifying cells, it means that one sister is more likely to exit the proliferative niche of the transit-amplifying compartment and become post-mitotic. For simple columnar epithelia, it is possible that post-mitotic separation provides a cellular mechanism for the neutral drift that governs stem cell population dynamics. All intestinal cells have a similar probability of undergoing post-mitotic separation, allowing one daughter to remain in its current niche position and the other to leave. It is unlikely that post-mitotic separation always produces a heterogeneous cell pair, as this would only readily occur near a niche boundary. However, this mechanism could influence overall homeostasis and protect stem cell number by slowing neutral drift (i.e. ensuring that one daughter remains close to its birth place, making it more likely to remain in a proliferative niche) (Fig. 4J).

The reason for why one daughter cell can remain closer to its place of birth may be related to the relative ease of movement in different parts of the crypt. For instance, it is likely that movement is easier in the linear region of the transit-amplifying compartment than in the curved, highly packed crypt base (Quyn et al., 2010). The upper end of the curvature of the crypt base in the small intestine is characterised by a narrowing that could act as a restriction point. This means that if cells are born near this restriction point (the stem-cell–transit-amplifying compartment boundary), and separate so that one is placed above and one below this point, the former is much more likely to move than the latter.

Reduced post-mitotic separation in *Apc* mutant cells provides an explanation for their increased probability to colonise a niche (Vermeulen et al., 2013; Baker et al., 2014). Neither mutant sister is likely to be displaced from its birthplace, instead, they remain in close proximity to each other. Together with their well-characterised decreased migration, which we confirmed in organoids (Fig. 3D), this could significantly decrease the number of *Apc* mutant cells exiting proliferative compartments (Nelson et al., 2012). As a result, in *Apc* mutant epithelia, many sisters would remain in a proliferative niche, resulting in increased stem cell numbers. This explains the increased number of cells in the crypt base of *Apc^Min/+^* tissue (Quyn et al., 2010). A reduction in post-mitotic separation and decreased migration may confer on *Apc* mutant cells the competitive advantage that causes their preferred niche retention (Fig. 4) (Nelson et al., 2012). Changes in the positioning of wild-type and *Apc* mutant cells could also be responsible for the measurable differences in *Apc^Min/+^* tissue that appears histologically normal. The decrease in the regularity of crypt shape and packing that is detectable by high-resolution optical imaging and high-frequency ultrasound could reflect altered post-mitotic placement of cells and could be caused by increased retention of expanding clones of *Apc^Min/Min^* mutant cells in *Apc^Min/+^* tissue (Fatehullah et al., 2016a).

Post-mitotic placement is likely to contribute to crypt fission, the process that produces two daughter crypts and is responsible for elongation of intestinal tract (Humphries and Wright, 2008). Initiation of crypt fission involves the formation of a cluster of stem cells at the crypt base, which marks the point of bifurcation (Langlands et al., 2016). Dynamic post-mitotic rearrangements of daughters could explain how these clusters form. We found that in many cases, mitoses next to Paneth cells resulted in separating sisters. The tight packing at the crypt base and the larger size and stiffness of Paneth cells means that once mitotic daughters of a dividing stem cell at the crypt base remain adjacent to each other, it is increasingly difficult for daughters of subsequent divisions to separate due to the physical constraint generated. This could cause the initial clustering of Lgr5-positive cells marking the initiation of fission.

Many mechanisms might contribute to post-mitotic separation. However, our data suggest that INM and the ability to asymmetrically segregate basal processes play an important role. A role for APC in INM, as suggested by our data, is consistent with findings in neuro-epithelia where loss of *Apc* disrupts INM (Ivaniutsin et al., 2009). In the neuro-epithelium, INM relies on microtubules for nuclear movement and actomyosin activity for cell rounding (Spear and Erickson, 2012; Xie et al., 2007). In the intestinal epithelium, INM may also involve microtubules. Specifically, the apical-basal microtubule scaffold may facilitate the nuclear movement during INM (Fig. S1). APC regulates both microtubules and actin (Näthke et al., 1996; Zumbrunn et al., 2001; Okada et al., 2010), and cytoskeletal defects resulting from *Apc* mutation could compromise microtubule bundles. We observed a distinct microtubule bundle in cells in early mitosis during INM (Fig. S1) suggesting that microtubules may contribute to the nuclear movement during this process. The number of microtubules in large parallel arrays is significantly reduced in *Apc^Min/+^* cells in the inner ear and such changes in the microtubule arrays in intestinal epithelial cells could compromise the efficiency of INM and reduce sister separation (Mogensen et al., 2002). Disruption of the microtubule scaffold may also add to defects in cell volume and height as observed in *Apc^Min/Min^* cells and as suggested by a recent report showing that disruptions of the apical-basal orientation of microtubules can reduce cell height (Toya et al., 2016).

APC is also involved in the nucleation of actin filaments (Okada et al., 2010). In this context the finding that actin rearrangements in mitosis that facilitate cell shape changes contribute to post-mitotic separation in the intestinal epithelium is particularly intriguing and provides a possible explanation for the defects we found in *Apc* mutant epithelia (McKinley et al., 2017, preprint). Recent work has demonstrated that the ability of APC to nucleate actin assembly is crucial for the disassembly of focal adhesions (Juanes et al., 2017). The turnover of focal adhesions is important for directed cell migration and may also be involved in partial detachment of the basal membrane and cell shape changes during INM. Defective disassembly of focal adhesions may contribute to the reduced speed of INM and the decreased movement of *Apc* mutant cells, and prevent post-mitotic separation.

The formation and position of the basal process underlies post-mitotic separation and we demonstrate that asymmetric process localisation actively promotes neighbour exchange and niche exit. How basal processes form is unclear, whether as a cause or a consequence of mitosis. In *Apc* mutant cells in the small intestine, as previously shown in the colon (Bellis et al., 2012), processes are usually symmetrically placed and we found that they form more slowly. The increased time required to complete INM in *Apc* mutant cells (Fig. 6) may be responsible, by reducing the time available to establish an asymmetric process.

Cell morphology is also important for post-mitotic separation. Cells in highly abnormal regions of FAP tissue were significantly compressed laterally, suggesting that *Apc* mutant cells are also smaller than wild-type cells in human tissue. Reduced cell volume could cause lateral and/or apical-basal compression as well as restricting nuclear movement and impairing INM. Consistent with this idea, we found several examples of mitotic cells that were some distance from the apical surface in transformed human intestinal tissue (Fig. 6H). A lack of mitotic sister separation could cause mutant cells *in vivo* to remain close together and colonise a niche more successfully than wild-type cells. Altered cell morphology is evident in human intestinal organoids after *Apc* depletion and is also seen upon mutation of KRAS, p53 or SMAD4 (Drost et al., 2015), suggesting that post-mitotic separation could also be compromised by other mutations that affect cell morphology.

In summary, we provide evidence that post-mitotic separation is a general mechanism used by intestinal epithelial cells to control niche access. This cellular mechanism could further explain the stochasticity of intestinal homeostasis and how it becomes biased to create a pre-neoplastic state.

## MATERIALS AND METHODS

### Mice

All experiments involving mouse tissue were performed under UK Home Office guidelines. CL57BL/6 wild-type, Lgr5-EGFP-IRES-creERT2 (*Lgr5^GFP/+^*), *Apc^Min/+^* and R26-rtTA Col1A1-H2B-GFP (H2B-GFP) mice were killed by cervical dislocation or CO_2_ asphyxiation.

### Tissue preparation – mouse small intestine

Adult mouse small-intestine was washed briefly in phosphate-buffered saline (PBS) and fixed with 4% paraformaldehyde (PFA) for 3 h at 4°C. Intestine was cut into 2×2 cm^2^ pieces and fixed in 4% PFA overnight at 4°C. The tissue was embedded in 3% low-melting-point agarose and sectioned at 200 µm intervals using a Vibratome (Leica). Cut sections were washed in PBS and permeabilised for 2 h with 2% Triton X-100 and incubated with blocking buffer [1% bovine serum albumin (BSA), 3% normal goat serum and 0.2% Triton X-100 in PBS] for 2 h at 4°C. Tissue was incubated for 48 h with Hoechst 33342 (ThermoFisher, 1:500) and phalloidin (Molecular Probes, 1:150) diluted in working buffer (0.1% BSA, 0.3% normal goat serum and 0.2% Triton X-100 in PBS) at 4°C. The tissue was washed with PBS before mounting in Prolong Gold (ThermoFisher). Sections were mounted on coverslips between two 120 µm spacers to preserve tissue structures.

### Organoid culture

Organoids were generated from mouse small intestinal crypts as previously described (Sato et al., 2009). Briefly, small intestines were removed and washed in PBS and opened longitudinally. Villi were removed by scraping the lumenal surface with a coverslip. Tissue was washed in PBS, incubated in 30 mM EDTA (20 min) and crypts dislodged by vigorous shaking. Crypt suspensions were centrifuged (72 ***g***, 4°C) and the pellet washed twice in PBS and dissociated to single cells with TripLE Express (Life Technologies) at 37°C for 5 min. Cells were resuspended in Advanced DMEM/F12 (ADF) and filtered through a 40 µm cell strainer (Greiner). Single cells were resuspended in growth factor-reduced Phenol Red-free Matrigel (BD Biosciences). Organoids were grown in crypt medium [ADF supplemented with 10 mM HEPES, 2 mM glutamax, 1 mM N-acetylcysteine, N2 (Gemini), B27 (Life Technologies), penicillin-streptomycin (Sigma-Aldrich), growth factors (EGF, 50 ng/ml, Invitrogen; Noggin, 100 ng/ml, eBioscience)] and R-Spondin conditioned medium (1:4). Chiron99021 (3 µM; Invitrogen), valproic acid (1 mM; Invitrogen) and Y27632 (10 µM; Cambridge Bioscience) were added to organoids for the first 48 h. Organoids were passaged by physically breaking up Matrigel, washing in ADF, dissociation by pipetting and reseeding in Matrigel.

### Human tissue

Human tissue used in this study was the same as used for a previous study (Fatehullah et al., 2016a). All tissue collected was approved by the Tayside Tissuebank subcommittee of the Local Research Ethics Committee from consenting donors and obtained in accordance with approved guidelines and according to the principles expressed in the Declaration of Helsinki. FAP biopsies from one FAP patient was obtained during routine colonoscopy surveillance.

### Organoid immunofluorescence

Organoids were grown in eight-well chamber slides (Ibidi) for 1–2 days at 37°C in 5% CO_2_. Organoids were fixed with warmed 4% PFA in PBS (pH 7.4) for 30 min (37°C), permeabilized for 1 h in 1% Triton X-100 (this and subsequent steps were performed at room temperature), blocked for 1 h (1% BSA, 3% normal goat serum and 0.2% Triton X-100 in PBS). Organoids were incubated overnight in primary antibodies diluted in working buffer (0.1% BSA, 0.3% normal goat serum and 0.2% Triton X-100) [γ-tubulin (Sigma, T6557, 1:500), GFP (Abcam, ab13970, 1:500); β4-integrin (Abcam, ab25254, 1:100); tubulin, (Abcam, ab6160, 1:200)], washed five times with working buffer before overnight incubation with secondary antibodies diluted in working buffer [Alexa Fluor-conjugated (1:500, Molecular Probes)] along with 5 µg/ml Hoechst 33342 and Alexa Fluor-conjugated phalloidin (1:150). Organoids were mounted in Prolong Gold overnight.

### Microscopy

Images of tissue and organoids were acquired with a Zeiss LSM 710 or LSM 880 with Airyscan (Carl Zeiss) using 25× or 40× Zeiss objective lenses and immersion oil with refractive index of 1.514. Serial image stacks were acquired with an optical section size of 0.8 μm.

### Confocal live imaging

Organoids were grown in Matrigel and spread thinly onto 35 mm^2^ glass-bottom dishes (World Precision Instruments). Crypt medium was supplemented with 2 mg/ml doxycycline to induce H2B–GFP expression. For live-cell imaging of mitochondrial dynamics, induced H2B–GFP organoids were incubated with 500 nM Mitotracker DeepRed FM (ThermoFisher Scientific) in crypt medium for 1 h at 37°C and 5% CO_2_. Subsequently, staining medium was replaced with fresh crypt medium containing growth factors. For live imaging of actin, organoids were stained with 100 nM SiR-Actin in crypt medium overnight. Organoids were placed in a live-cell imaging chamber attached to a Zeiss 710 confocal microscope maintained at 37°C and 5% CO_2_. Images were acquired using a 40× Zeiss immersion objective. Serial image stacks were acquired at optimal interval sizes using minimal laser power every 6 min.

### Spindle angle measurements

Spindle orientation was measured using image stacks and analysed with the Imaris imaging software (Bitplane). Surfaces of the Hoechst 33342, γ-tubulin and phalloidin signals were rendered using the isosurface tool. Similar to a report in the mouse colon (Bellis et al., 2012), we used two angles to represent spindle orientation: (1) relative to the crypt axis (axial angle) and (2) relative to the apical surface (apical angle). The apical surface was defined using the F-actin signal at the lumenal surface. To calculate angles, three sets of measurement points were manually placed in 3D: (1) two points defining the two centrosomes. The connection between them represents the spindle axis; (2) two points placed at either end of the crypt so that the axis formed between them is representative of the crypt axis; and (3) three points placed on the rendered phalloidin surface to represent the cells' apical surface.

Axial angles are the angles between the spindle and crypt-axis. The apical angle is calculated by using the three measurement points to determine the normal surface vector to the apical plane. The axial angle between this defined apical surface and the spindle axis was calculated as below.

#### Calculating the axial angle

The spindle-axis vector (s) is calculated by using centrosome point 1, (*S*_*x*1_, *S*_*y*1_, *S*_*z*1_), and centrosome point 2 (*S*_*x*2_, *S*_*y*2_, *S*_*z*2_) (Eqn 1). The crypt-axis vector (c) is calculated using crypt-axis point 1 (*C*_*x*1_, *C*_*y*1_, *C*_*z*1_), and crypt-axis point 2 (*C*_*x*2_, *C*_*y*2_, *C*_*z*2_) (Eqn 2).(1)
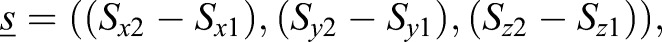
(2)
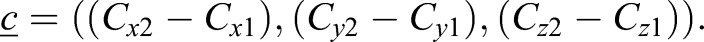
The α-angle is calculated by projecting the vector s on vector c (Eqn 3):(3)
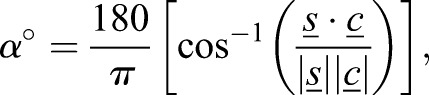
where:(4)
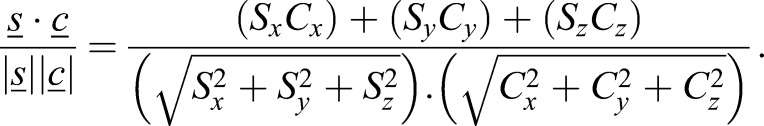


#### Calculating the apical angle

The apical angle is calculated by using three apical surface points (AP): (*A*_*x*_, *A*_*y*_, *A*_*z*_), (*B*_*x*_, *B*_*y*_, *B*_*z*_) and (*C*_*x*_, *C*_*y*_, *C*_*z*_), placed on the plane, which approximates the apical surface. The co-ordinates of these points are used to determine two vectors, a and b (Eqns 5 and 6):(5)
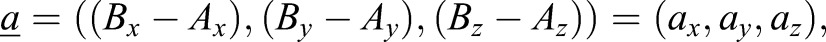
(6)
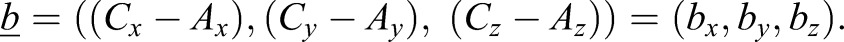
These vectors can subsequently be used to determine the normal surface vector (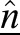) to the apical plane by finding the cross product between vectors a and b (Eqn 7):(7)
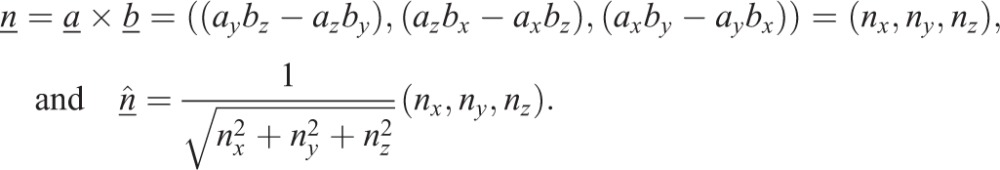
The normal surface vector can then be used to determine the angle between the spindle vector (s) and the surface plane (Eqn 8):(8)
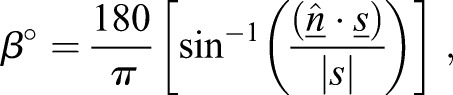


given that 
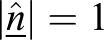
.

### INM measurements

Apical and basal interkinetic nuclear migration was measured by determining the distance between the nucleus and the plane of the epithelium. The plane of the epithelium was defined as the plane formed between the basal nuclear centres of neighbours surrounding the query nucleus.

To find the absolute distance from the mitotic cell, *M*(*x*_0_, *y*_0_, *z*_0_) to the epithelial plane, *P*(*Ax*+*By*+*Cz*+*D*=0), the epithelial plane was defined by the plane formed between three neighbouring epithelial cells:

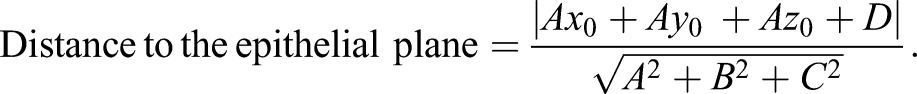

Distance measurements were calculated for ten planes encompassing each permutation of neighbouring cells. The average distance for these ten planes was taken as the representative distance of the query cell in reference to the plane of the epithelium in which it originated. This was to account for variability induced by the curvature within the organoid branches.

### Definition of stem cell and transit-amplifying compartments

Stem cell and transit-amplifying compartments were defined based on the average position of Lgr5-positive cells along the crypt–villus axis as measured in Lgr5–GFP organoids (Fig. S5). As Lgr5 can also be expressed in the early transit-amplifying compartment (Quyn et al., 2010), we conservatively defined the stem cell compartment based on the average position of Lgr5-positive cells rather than the average distance of the Lgr5-positive cell furthest from the crypt base. The number of stem and Paneth cells is similar between intestinal crypts and organoids (Langlands et al., 2016; Martinez Rodriguez et al., 2012).

### Distance measurements

Cell position was determined by placing a measurement point in the centre of each nucleus. The co-ordinates of individual points were used to measure the distances between nuclei or from the crypt base. The crypt base was defined by a reference nucleus manually chosen at the crypt base. The reference nucleus was determined at each time point. Distance between points was calculated by using the standard formula:
(9)
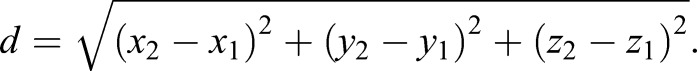


### Time-lapse analysis and scoring

Time-lapse image stacks were analysed manually with Imaris (Bitplane). All mitotic events were marked by using the ‘3D spots function’. Time-point 0 was denoted as the time-point immediately before cytokinesis. Each mitotic daughter cell was tracked until its death, subsequent division or exit from the imaging window. If daughter cells were separated by a neighbouring nucleus after basal INM (at 120 min) they were scored as separated. Displacement (a measure of velocity over a given time interval) from the ‘point of birth’ was calculated as the change in distance between the start (mitotic mother) and end position of a daughter, divided by the time interval. Daughter 1 was always classified as the daughter closest to the base of the crypt.

### INM measurement

We determined the distance covered by nuclei during INM by measuring the distance of the mitotic nucleus to a defined basal reference plane. The basal reference was defined as the plane formed by neighbouring interphase cells. The co-ordinates of five neighbouring nuclei most proximal to the mitotic cell were determined. These interphase cells often did not form a simple plane due to the curvature of the epithelial sheet. To account for the shape, we calculated the nearest distance from the position of the mitotic cell to the planes formed by combinations of each of three nuclei for the five neighbours. The average distance from these (ten) planes was then used as the estimated apical distance travelled during INM (Fig. S5). Please refer to supplementary information for further details. (Fig. S5D and corresponding legend)

### Cell height measurement

Apical-basal distance was measured as the distance between the apical and basal surfaces with Imaris. Distances were calculated in the optical section at the centre of the chosen cell by recording one 3D measurement point in the middle of the apical and one in the middle of the basal surface. The distance between these points was the apical-basal distance.

### Sample size and statistical analysis

Analyses were performed using at least three different organoids. Individual cell comparisons were performed using at least ten cells. Comparisons had to be made between organoids imaged in separate imaging sessions because each time-lapse took 3 days. All statistical tests were performed using Prism 6.0a (GraphPad). Tests were performed as described in figure legends (significance: ns, not significant, *P<0.05, **P<0.01, ***P<0.001, ****P<0.0001).

### Multicellular computational model

All simulations were undertaken in the Chaste framework (Mirams et al., 2013). We extended the model presented in Dunn et al. (2016) to permit variable separation of cells after division. In summary, cells are represented by their centres, which are free to move on a surface (in 3D space) that is defined by using measured crypt geometry. Cells move because of forces exerted on them due to compression of, and by, neighbouring cells. We are using the optimal model identified in Dunn et al. (2016) – Model 6. In this model, cells divide after a uniformly distributed time that depends on the level of Wnt (imposed as a linear gradient) experienced when the parent cell divided. Additionally, if cells are compressed beyond a given threshold they pause in the G1 phase of the cell cycle. All parameters used are as described in Dunn et al. (2016). As in previous 3D models of the crypt (Dunn et al., 2016, 2013), cell division occurs in a direction uniformly drawn from the sphere surrounding the centre of the dividing cell. Daughter cells are placed at a specified distance from one another. Previously, in all existing models, this distance is chosen so daughter cells are adjacent to each other. We modified this parameter so that two thirds of all cell divisions resulted in the daughter cells being placed next to each other (a separation of one cell diameter), the remaining third resulted in the daughter cells being separated by *S* cell diameters. We vary this parameter between 1 and 4 to measure the effects on separation of cells in the virtual crypt.

## Supplementary Material

Supplementary information
