# Supplementary material for: Interkinetic nuclear migration and basal tethering facilitates post-mitotic daughter separation in intestinal organoids
Source: J Cell Sci. 2017 Nov 15;130(22):3862–77. doi: 10.1242/jcs.211656 (PMC5702049; doi:10.1242/jcs.211656)
Supplement: Supplementary information [file joces-130-211656-s1.pdf]

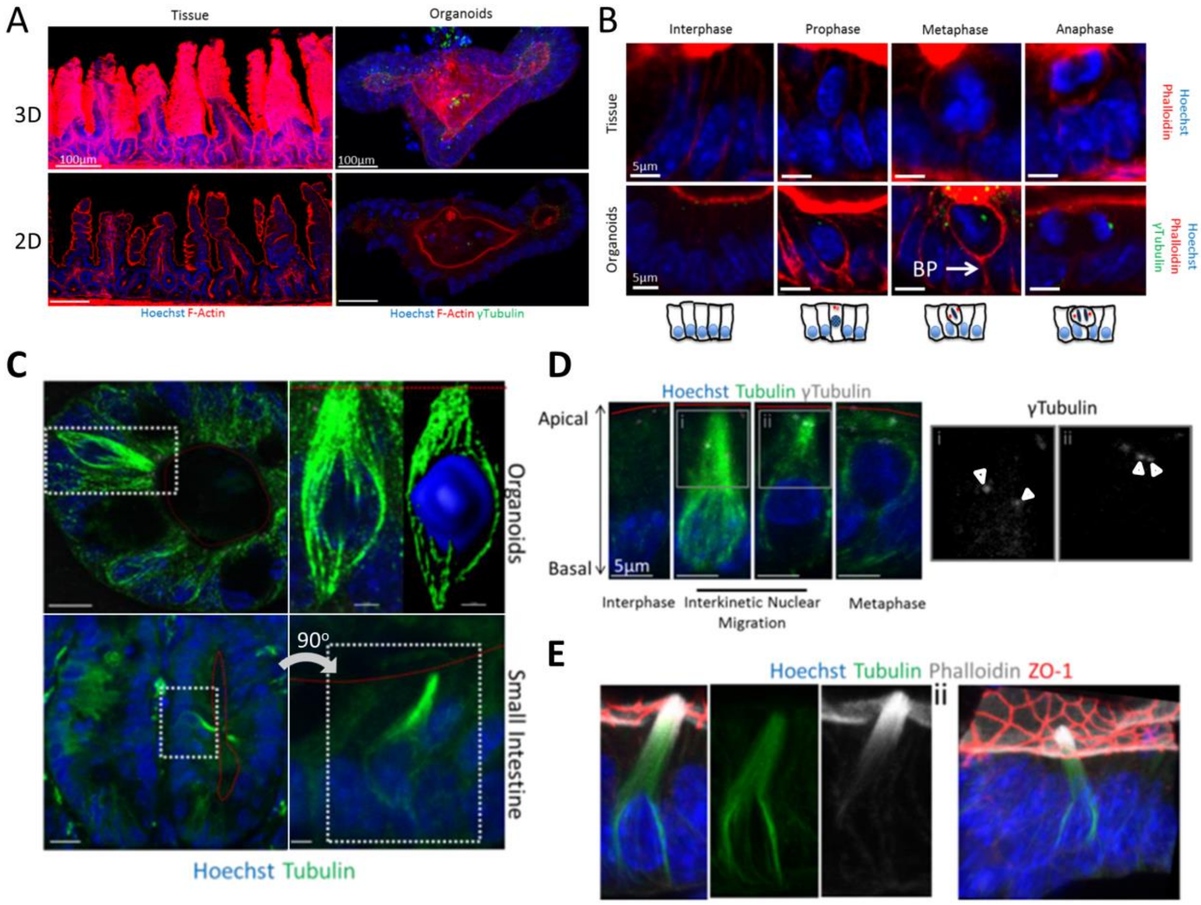

Carroll et al., S1 Figure

Figure S1 . Mitosis in intestinal tissue andintestinal organoids.

(**A**) Maximum intensity projection (3D) and sections (2D) of a vibratome section of mouse small-intestine (tissue) and an intestinal organoid stained with Hoechst (blue), phalloidin (red), and γ-tubulin (green).

(**B**) Sections (2D) of mitotic stages visualised in intestinal crypts of whole tissue and organoids. Interphase cells maintain basally positioned nuclei. During mitosis the apical cell surface remains aligned with neighbouring cells. Chromatin condensation occurs during prophase and the nucleus is displaced apically. During INM, the rounded mitotic cell remains attached to the basal membrane by a basal process (BP). After alignment with the apical surface the metaphase plate forms apically and is directly followed by anaphase in which cells have two clear sets of sister chromatids. The images in the bottom panel are shown for reference and are the same as those shown in Figure 1A.

(**C**) An intestinal organoid (top panels) and small intestinal tissue (bottom panels) stained with Hoechst (blue) and an antibody against tubulin (green). In the top right panels , surface rendering reveals the structure of an apical-basal array of microtubules. The apical surface is marked by a red dashed line. The mitotic cell outlined by a white dashed box in the left bottom panel, is shown enlarged and rotated in the right bottom panel.

(**D**) Representative examples of mitotic cells in organoid epithelium at stages of interkinetic nuclear migration. Microtubule polymerisation is most evident when nuclei are basally localised. As nuclei move apically, microtubule polymerisation is mostly at the apical-most side of the microtubule scaffold. There was no detectable microtubule polymerisation in rounded up mitotic cells. Indicated cells are not differentiated due to the detectable pair of centrosomes (white arrows in i and ii). The apical surface is marked by a red dashed line.

(**E**) A tuft cell in an intestinal organoid. Organoids were stained with Hoechst (blue), phalloidin (white) and antibodies against tubulin (green) and ZO-1 (red) displayed as a section (i) or in 3D (ii). Tuft cells have a ‘tuft’ of microvilli that protrude apically into the lumen. They are fully differentiated, predominantly found within the differentiated zone, and lack detectable centrosomes.

Journal of Cell Science • Supplementary information

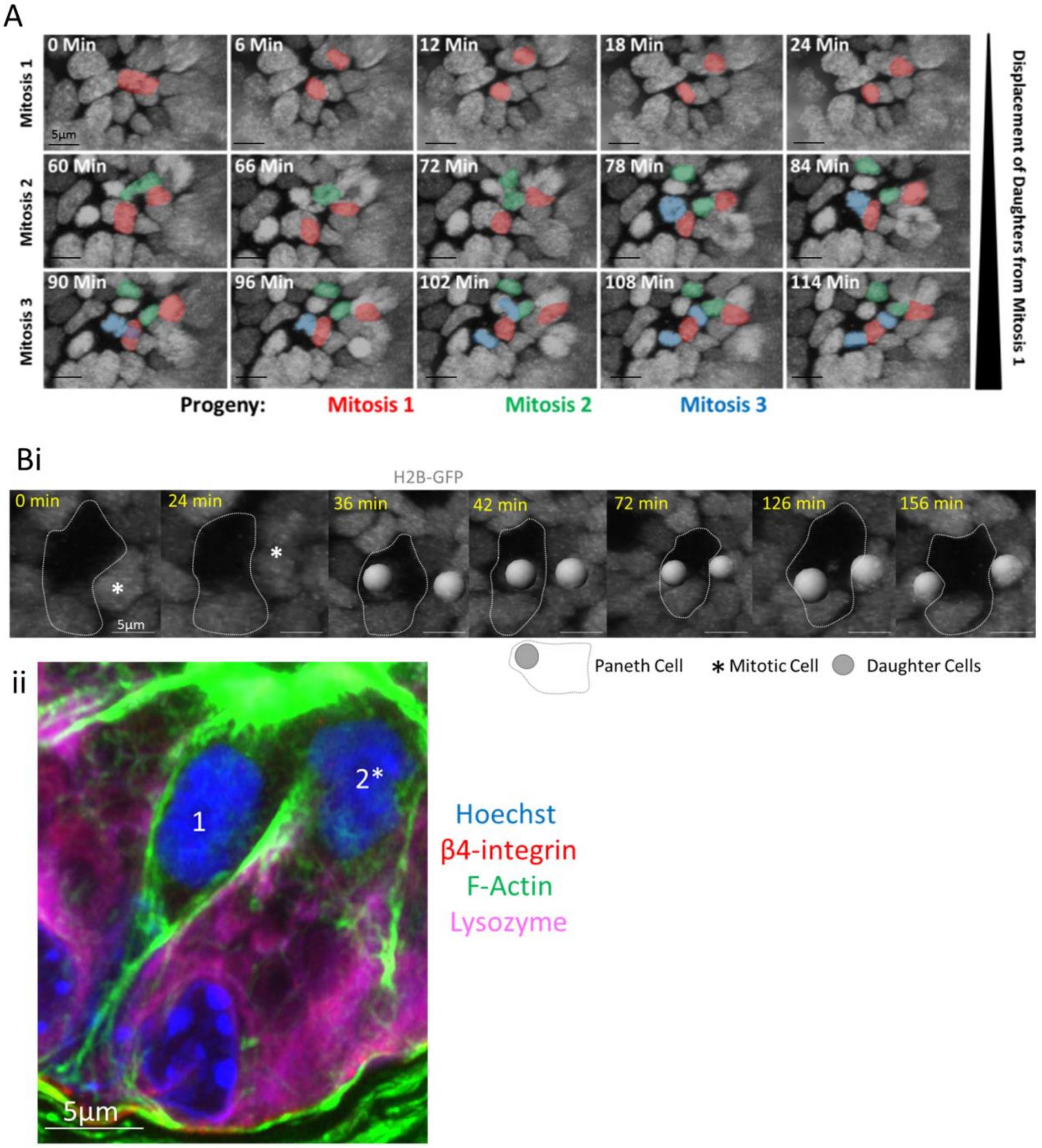

Carroll et al., S2 Figure

Journal of Cell Science • Supplementary information

Figure S2. Alternative methods of separation

(**A**) Separation of daughters is enhanced by movement of neighbouring mitotic cells. Displayed are 3D projections of the movements of the progeny of a mitotic cell (Original Mitosis, [Mitosis 1; red]) and the progeny of two neighbouring mitotic cells (Mitosis 2, green; Mitosis 3; blue). Time 0 marks metaphase of the original mitotic cell.

(**B**) **i**)Live imaging of a wild-type H2B-GFP organoid. A Paneth cell can be clearly identified based on morphology and distribution of neighbouring nuclei (dashed line). A mitotic cell (white stars) proximal to the Paneth cell divides to produce two daughters (white balls) who separate and then renter the epithelial plane adjacent to the Paneth cell. **ii**) Fixed image of small-intestinal tissue, stained with Hoechst (Blue), β4-Integrin (red), lysozyme (magenta) and phalloidin (green). A recent mitosis produced two daughter cells (1, 2*) which have become separated and have reinserted on either side of the Paneth cell. The image is a duplicate of Figure 5A, showing lysozyme staining.

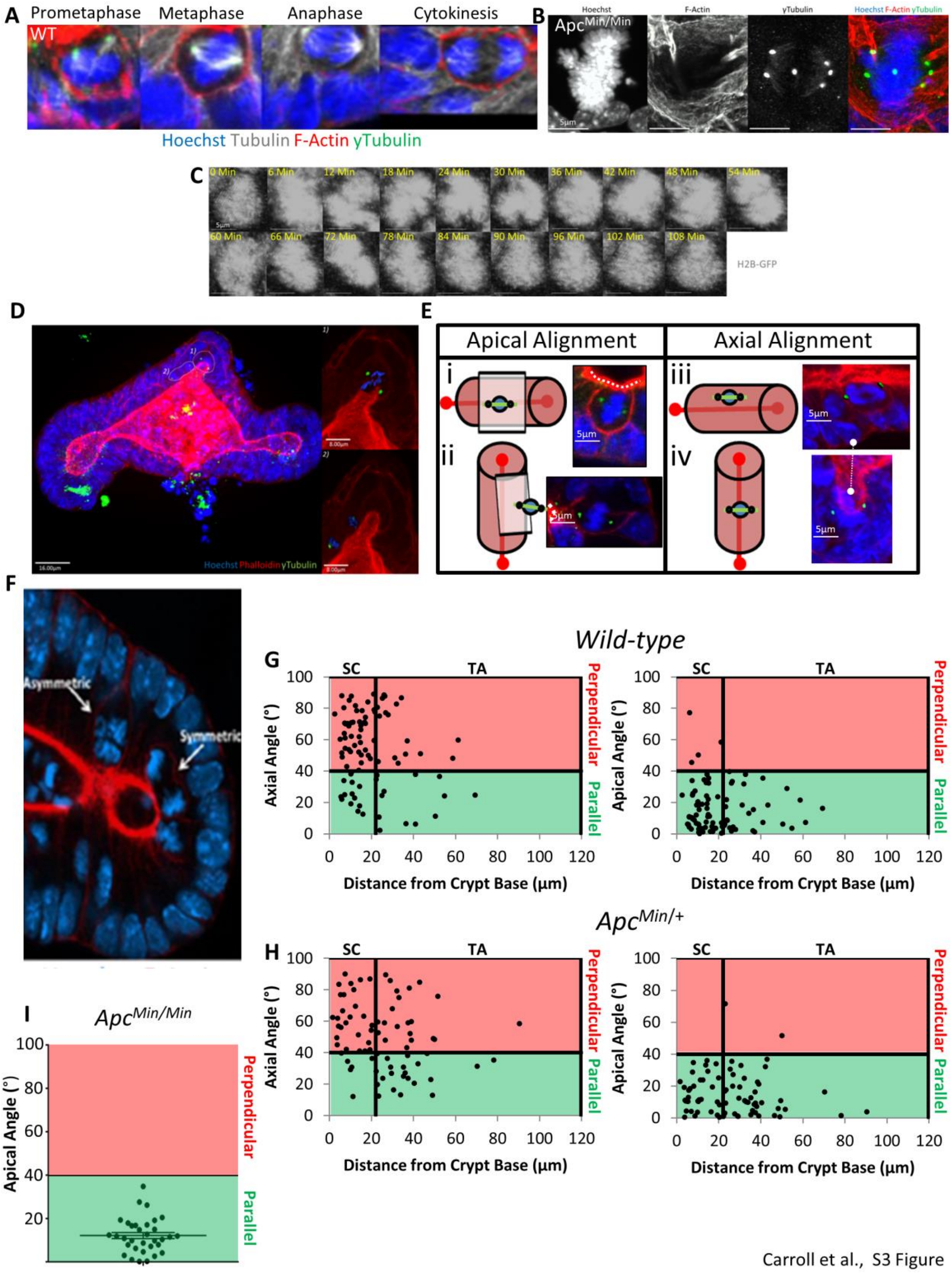

Carroll et al., S3 Figure

Journal of Cell Science • Supplementary information

Figure S3. Spindle orientation in intestinal organoids

(A) Representative mitotic cells in prometaphase, metaphase, anaphase and during cytokinesis. Organoids are stained with Hoechst, phalloidin and antibodies against tubulin and γ-tubulin to visualise nuclei (blue), F-actin (red), microtubules (white) and centrosomes (green). Centrosomes are located equidistantly on either side of the metaphase plate once it is fully established.

(B) A representative example of an $Apc^{Min/Min}$ mitotic cell with a multipolar spindle. $Apc^{Min/Min}$ organoids were stained with Hoechst (blue), phalloidin (red) and an antibody against γ-tubulin (green) to visualise DNA, F-actin and centrosomes.

(C) A representative example of an $Apc^{Min/Min}$ cell undergoing mitotic slippage. Displayed are stills from live-imaging of an $Apc^{Min/Min}$ H2B-GFP organoid. Chromosome condensation is clearly observed as the cell enters prophase. Instead of proceeding with mitosis, chromosomes de-condense as the cell returns to interphase.

(D) A representative example of a wild-type organoid stained with Hoechst (blue), phalloidin (red), γ-tubulin (green) to visualise DNA, F-actin and centrosomes. Two mitotic cells are highlighted, one in metaphase (top) and one in anaphase (bottom). Surface rendering in Imaris can clearly highlight individual cells and their two centrosomes.

(E) Potential spindle alignments in intestinal organoids. Spindle orientation was determined in reference to: 1) the axis of tissue growth; the crypt-villus axis or 2) the apical surface. Spindle orientations are the angle between the spindle and crypt-villus axis (Axial angle) or apical surface (Apical angle). Examples of each type of spindle alignment is displayed; i) Parallel to the crypt-villus axis (Crypt lengthening), ii) Perpendicular to the crypt-villus axis (Crypt widening), iii) Parallel to the apical surface (‘symmetric’ division) or perpendicular to the apical surface (‘asymmetric’ division). Reference axes are highlighted by the white dashed line. The pink rectangle in panels i and ii represents the plane of the apical surface.

(F) Representative example of an asymmetrically and symmetrically oriented division in the crypt base of an intestinal organoid. The organoid is stained with Hoechst (blue) and phalloidin (red). Note that a pro-daughter cell in the asymmetrically aligned mitoses is poised to inherit the basal process.

(G – I) Spindle orientations were determined for mitoses in G) wild-type, H) *Apc*$^{Min/+}$ and I) *Apc*$^{Min/Min}$ organoids. Only apical angles could be calculated for $Apc^{Min/Min}$ organoids due to loss of crypt-villus architecture. Data is displayed in reference to the crypt base. Angles greater than 40° were classified as perpendicular. Angles less than 40° are classified as parallel. Data is displayed as a function of distance along the crypt-villus axis. The stem cell compartment was defined as the curved region at the base of branches, approximately 20µm from the lumenal crypt base

Journal of Cell Science • Supplementary information

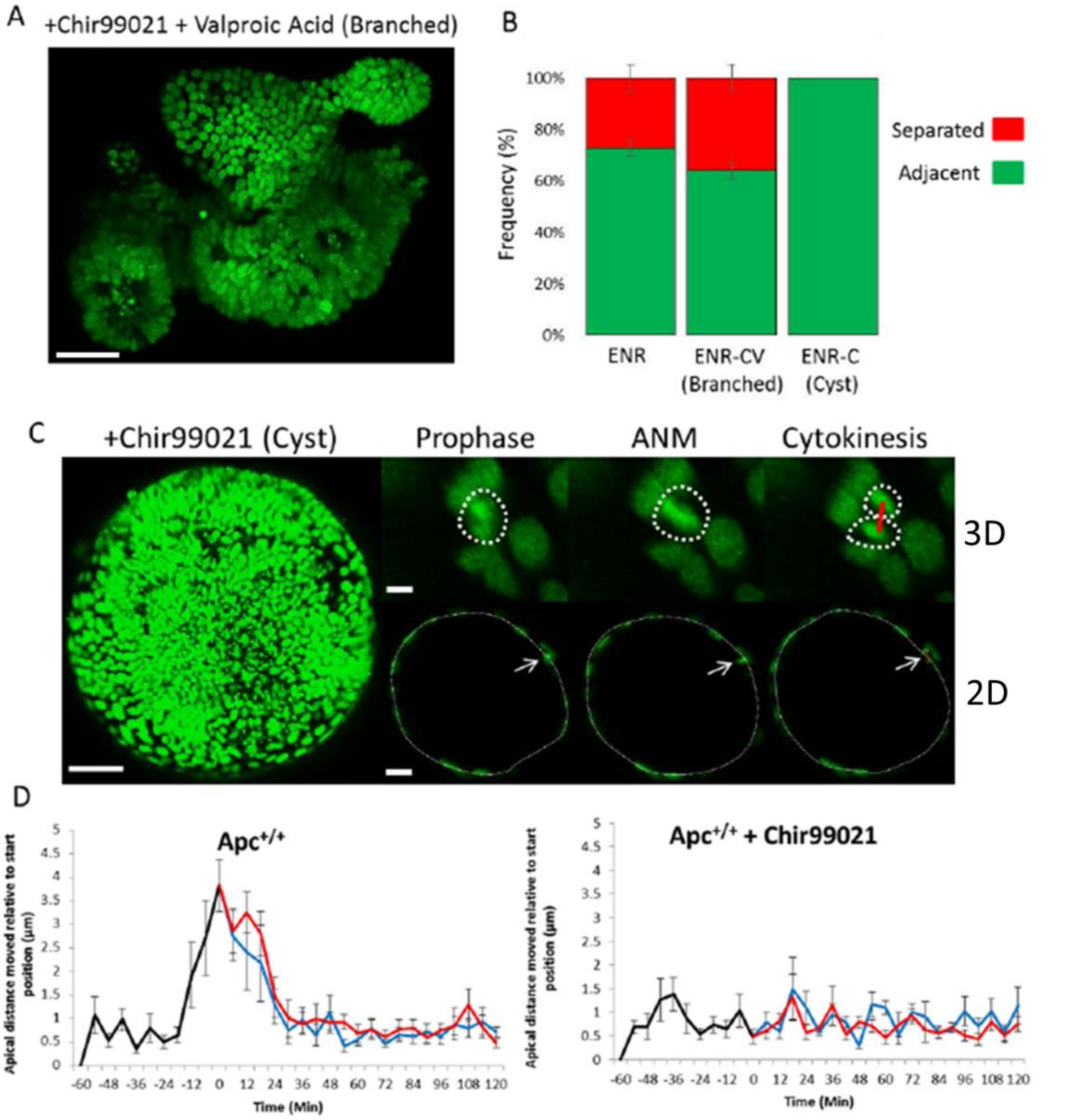

Carroll et al., S4 Figure

Journal of Cell Science • Supplementary information

Figure S4. Disruption of INM can be induced by chronic Chir99021 treatment

(A) An H2B-GFP intestinal organoid treated with Chir99021 and valproic acid. Treatment with Chir99021 and valproic acid has been shown to increase the frequency and distribution of Lgr5(+) cells along the crypt axis (Yin et al., 2014). Treated organoids retain crypt-villus architecture. Scale bar = 100µm

(B) Daughter cell placement after mitosis was scored in H2B-GFP organoids using time-lapse movies. Organoids were treated with Chir99021 and valproic acid to increase the stem cell content along the crypt-villus axis, or they were chronically treated with 10µM Chir99021 for 4 days to induce cyst formation in WT organoids. Division subtypes were compared to untreated organoids (ENR). Data was compared to the dataset in Figure 4B. (ENR N = 6 organoids, N = 491 mitoses; ENR-CV (branched) N = 3 organoids, N = 351 mitoses; ENR-C (cyst) N = 3 organoids).

(C) A wild-type H2B-GFP organoid chronically treated with 10µM Chir99021 for 4 days (left panel), scale bar = 100µm. Right panels highlight individual frames from live-recordings displaying a representative mitosis during prophase, apical interkinetic nuclear migration (ANM) and cytokinesis. 3D (maximum intensity projections,) and transversesections (2D) are shown. Scale bars = 5µm

(D) Dynamics of interkinetic nuclear migration during mitosis in Chir99021 treated organoids *were* measured relative to the starting distance (N = 10 cells). Data is displayed as mean +/- SEM. Measurements of the mother (black line) and daughter cells (red and blue lines) are superimposed. The wild-type dataset displayed is the same dataset as displayed in Figure 7D.

Journal of Cell Science • Supplementary information

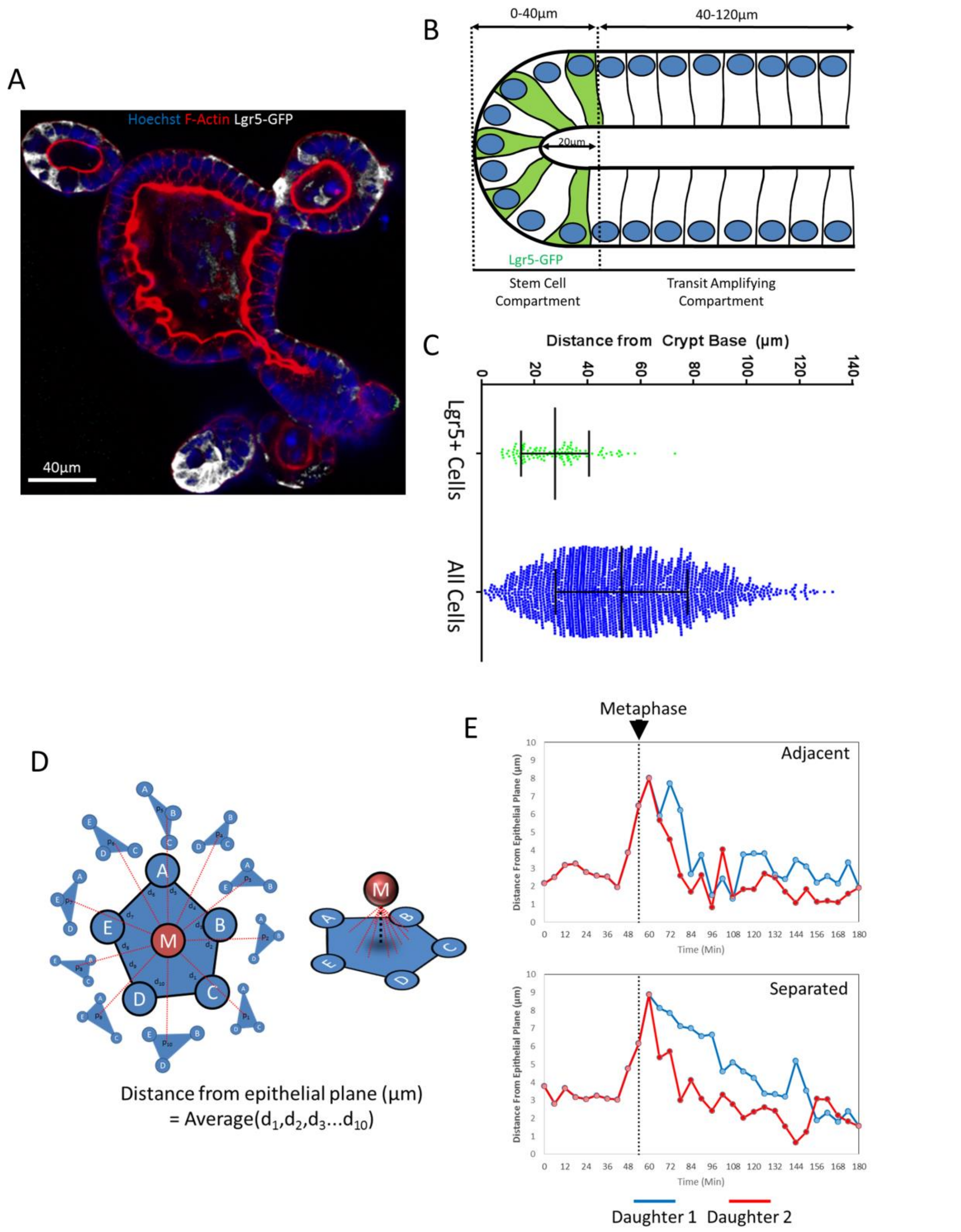

Carroll et al., S5 Figure

Journal of Cell Science • Supplementary information

Figure S5. Definition of tissue compartments and interkinetic nuclear migration

(**A**) An Lgr5-GFP expressing intestinal organoid stained with Hoechst (nuclei), phalloidin (F-actin) and GFP (Lgr5+ stem cells). Stem cells resided within the base of intestinal organoid branches, mostly residing within the curved region of the crypt base.

(**B**) The position of each Lgr5-GFP+ cell was recorded and compared to the positions of the total cell population with reference to the crypt base. Nuclear position was used as a surrogate for cell position and distances were compared to the nucleus closest to the base of the crypt. Data was pooled from 6 individual organoids. Data is displayed as mean +/- SD.

(**C**) Diagram showing the defined compartments within intestinal crypts. The majority of Lgr5+ stem cells were located approximately 0-40μm from the crypt base. This region was termed the stem cell compartment. This equated to the curved region at the base of the crypt, approximately 20μm from the lumenal crypt base. Above this region we defined as the transit-amplifying compartment. A small fraction of GFP+ cells resided above the defined stem cell compartment, similar to our previous studies in whole intestinal tissue.

(**D**) Interkinetic nuclear migration is quantified as the distance of the query cell in reference to the epithelial plane in which it originated. The plane of the epithelium is defined as the plane in which neighbouring nuclei are located. A plane is defined by the co-ordinates of 3 points. Therefore the distance was measured between the query cell and the plane formed by 3 of its neighbour nuclei. This process was repeated utilizing 5 neighbour cells. The average distance for each of these 10 planes was determined as distance from the epithelial plane.

(**E**) Examples of INM measurement for a cell undergoing adjacent placement (Adjacent) or post-mitotic separation (Separated). Distances were determined for each time-point during prophase and for each of the daughter cells (red and blue lines).

Journal of Cell Science • Supplementary information

Movies

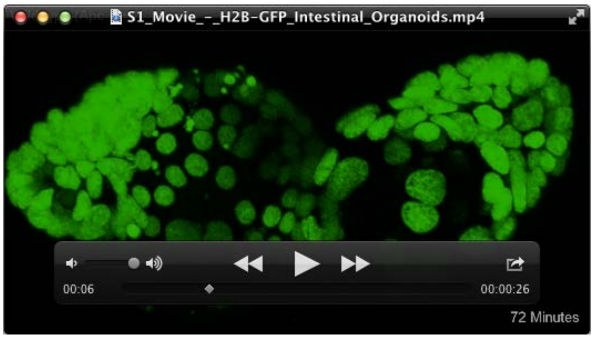

Movie S1. H2B-GFP Intestinal Organoids

Confocal LSM imaging of induced H2B-GFP organoids derived from wild-type and $Apc^{Min/+}$ mice (Both untransformed ($Apc^{Min/+}$) and transformed cysts ($Apc^{Min/Min}$)).

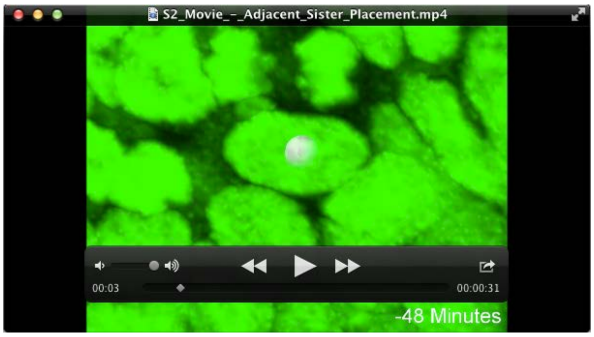

Movie S2. Adjacent Sister Placement

Confocal LSM imaging of an induced wild-type H2B-GFP organoid showing manual tracking of a mitotic cell and its progeny. Daughters were tracked manually using Imaris. In this example, both daughter cells 're-insert' into the epithelium as neighbours.

Journal of Cell Science • Supplementary information

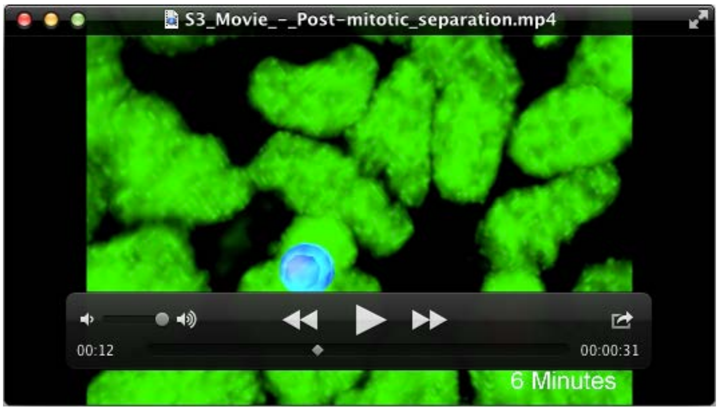

Movie S3. Post-mitotic Separation

Confocal LSM imaging of an induced wild-type H2B-GFP organoid showing manual tracking of a mitotic cell and its progeny. Daughters were tracked manually using Imaris. In this example, the 'blue' daughter cell is displaced from its sister.

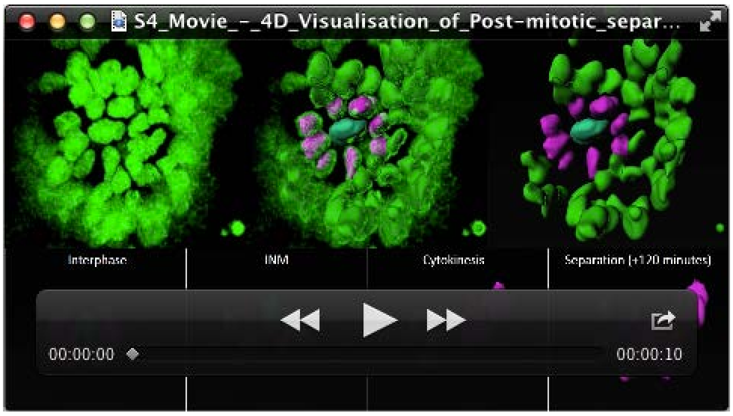

Movie S4. 4D Visualisation of post-mitotic separation

Confocal LSM imaging of an induced wild-type H2B-GFP organoid showing manual tracking of a mitotic cell and its progeny undergoing post-mitotic separation. Surface rendering was performed to highlight the mother (cyan), sisters (blue/red) and neighbour cells (magenta). The respective timelapse is shown in the top panels and a 3D rotation around the timepoints encompassing interphase, INM, cytokinesis and after separation are displayed in the bottom panels.

Journal of Cell Science • Supplementary information

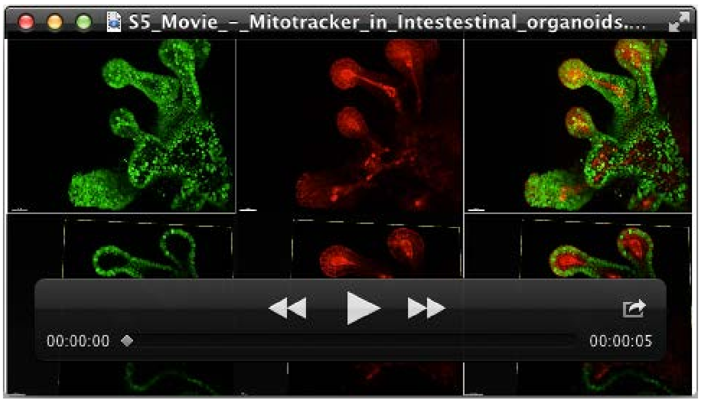

Movie S5. Mitotracker in Intestinal Organoids

Confocal LSM imaging of an induced H2B-GFP organoid treated with Mitotracker.

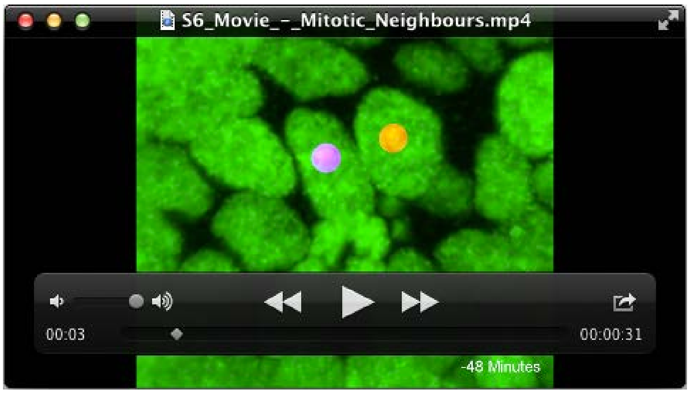

Movie S6. Mitotic neighbours

Confocal LSM imaging of an induced wild-type H2B-GFP organoid showing manual tracking of a mitotic cell and its progeny undergoing post-mitotic separation. In this example, the daughters of the original mitosis (red) are displaced by the placement of a daughter cell from an adjacent mitosis (purple).

Journal of Cell Science • Supplementary information

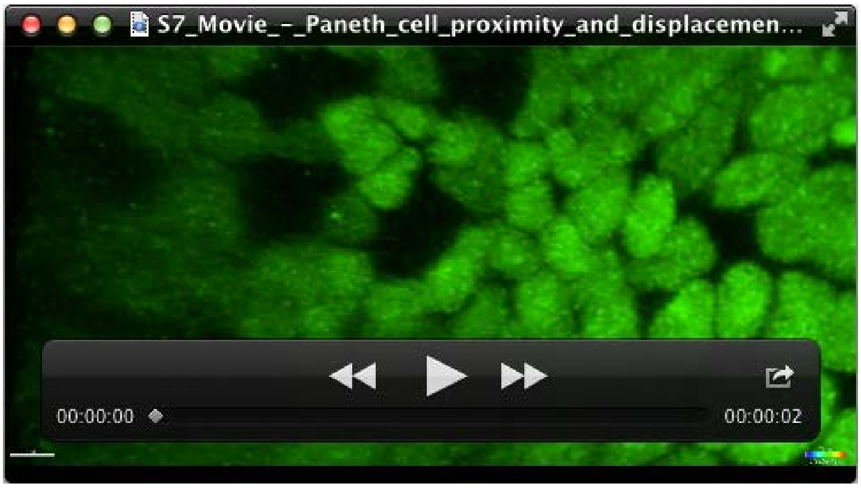

Movie S7. Paneth Cell proximity and displacement

Confocal LSM imaging of an induced wild-type H2B-GFP organoid showing manual tracking of a mitotic cell and its progeny undergoing post-mitotic separation. In this example, the daughters of this mitosis are displaced in proximity to a Paneth cell (recognisable by the large space with no nuclei). This movie was used in stills in S2 figure.

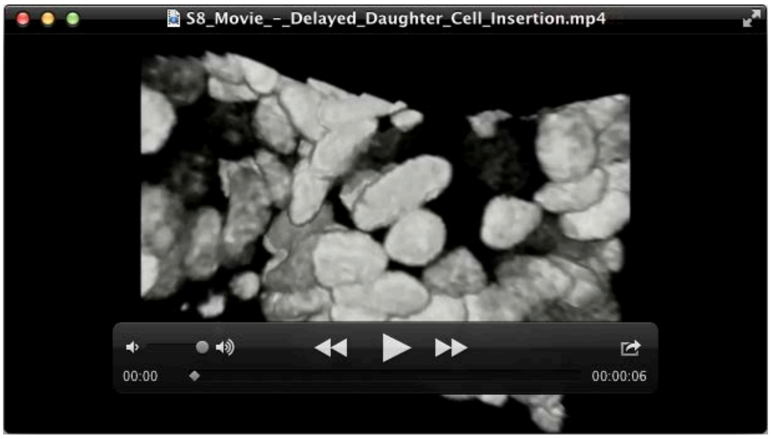

Movie S8. Delayed Daughter Cell Insertion

Confocal LSM imaging of an induced wild-type H2B-GFP organoid showing manual tracking of a mitotic cell and its progeny undergoing post-mitotic separation. In this example the left most daughter takes two attempts to reassume its interphase position, whilst the other is displaced. This movie was used for stills in Figure 5E.

Journal of Cell Science • Supplementary information

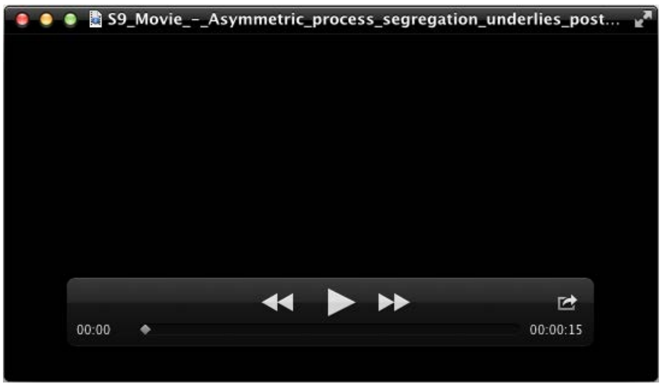

Movie S9. Asymmetric process segregation underlies post-mitotic separation

Confocal LSM imaging of an induced wild-type H2B-GFP organoid treated with SiR-Actin. The movie shows a mitotic cell undergoing post-mitotic separation in which one daughter retains the basal process. The two daughters (white spheres) are separated by a neighbour after reinsertion.

Journal of Cell Science • Supplementary information
